# Systems Biology Markup Language (SBML) Level 3 Package: Distributions, Version 1, Release 1

**DOI:** 10.1515/jib-2020-0018

**Published:** 2020-07-20

**Authors:** Lucian P. Smith, Stuart L. Moodie, Frank T. Bergmann, Colin Gillespie, Sarah M. Keating, Matthias König, Chris J. Myers, Maciek J. Swat, Darren J. Wilkinson, Michael Hucka

**Affiliations:** University of Washington, Seattle, USA; Eight Pillars Ltd, Edinburgh, UK; University of Heidelberg, Heidelberg, Germany; Newcastle University, Newcastle, UK; University College London, London, UK; Humboldt-University Berlin, Berlin, Germany; University of Utah, Salt Lake City, USA; QSP Simcyp, Sheffield, UK; California Institute of Technology, Pasadena, USA

**Keywords:** distributions, modeling, SBML, systems biology, uncertainty

## Abstract

Biological models often contain elements that have inexact numerical values, since they are based on values that are stochastic in nature or data that contains uncertainty. The Systems Biology Markup Language (SBML) Level 3 Core specification does not include an explicit mechanism to include inexact or stochastic values in a model, but it does provide a mechanism for SBML packages to extend the Core specification and add additional syntactic constructs. The SBML Distributions package for SBML Level 3 adds the necessary features to allow models to encode information about the distribution and uncertainty of values underlying a quantity.

***Corresponding author: Lucian P. Smith**, University of Washington, Seattle, USA, E-mail: lpsmith@uw.edu. https://orcid.org/0000-0001-7002-6386
**Stuart L. Moodie:** Eight Pillars Ltd, Edinburgh, UK, E-mail: stuart.moodie@eightpillars.uk.com. https://orcid.org/0000-0001-6191-5595
**Frank T. Bergmann:** University of Heidelberg, Heidelberg, Germany, E-mail: frank.bergmann@bioquant.uni-heidelberg.de. https://orcid.org/0000-0001-5553-4702
**Colin Gillespie and Darren J. Wilkinson:** Newcastle University, Newcastle, UK, E-mail: colin.gillespie@ncl.ac.uk (C. Gillespie), darrenjwilkinson@googlemail.com (D.J. Wilkinson). https://orcid.org/0000-0003-1787-0275 (C. Gillespie). https://orcid.org/0000-0003-0736-802X (D.J. Wilkinson)
**Sarah M. Keating:** University College London, London, UK, E-mail: s.keating@ucl.ac.uk. https://orcid.org/0000-0002-3356-3542
**Matthias König:** Humboldt-University Berlin, Berlin, Germany, E-mail: konigmatt@googlemail.com. https://orcid.org/0000-0003-1725-179X
**Chris J. Myers:** University of Utah, Salt Lake City, USA, E-mail: myers@ece.utah.edu. https://orcid.org/0000-0002-8762-8444
**Maciek J. Swat:** QSP Simcyp, Sheffield, UK, E-mail: maciej.swat@gmail.com
**Michael Hucka:** California Institute of Technology, Pasadena, USA, E-mail: mhucka@library.caltech.edu. https://orcid.org/0000-0001-9105-5960

SBML Level 3 Package Specification

## The Distributions Package for SBML Level 3

### Authors

Lucian P Smith
University of Washington
Seattle, WA, USA

Stuart L Moodie
Eight Pillars Ltd
Edinburgh, UK

### Contributors

Frank Bergmann
University of Heidelberg
Heidelberg,DE

Colin Gillespie
University of Newcastle
Newcastle, UK

Sarah Keating
University College London
London, UK

Matthias König
Humboldt University
Berlin, DE

Chris Myers
University of Utah
Salt Lake City, UT, USA

Maciej J Swat
QSP Simcyp
Certara, Sheffield, UK

Darren Wilkinson
University of Newcastle
Newcastle, UK

Michael Hucka
California Institute of Technology
Pasadena, CA, USA

Version 1, Release 1

April 2, 2020

The latest release, past releases, and other materials related to this specification are available at
http://sbml.org/Documents/Specifications/SBML_Level_3/Packages/distrib

*This* release of the specification is available at
https://co.mbine.org/specifications/sbml.level-3.version-1.distrib.version-1.release-1

## Contents

**1 Introduction and motivation** 5
1.1 What is the Distributions package? . . . 5
1.2 Scope . . . 5
1.3 This document . . . 5
**2 Background** 6
2.1 Problems with current SBML approaches . . . 6
2.2 Past work on this problem or similar topics . . . 6
2.2.1 Newcastle Proposal 2005 . . . 6
2.2.2 SBML Hackathon 2010: Seattle . . . 6
2.2.3 Statistical Models Workshop 2011: Hinxton . . . 7
2.2.4 HARMONY 2012: Maastricht . . . 8
2.2.5 COMBINE 2012: Toronto . . . 8
2.2.6 Package Working Group discussions 2013 . . . 8
2.2.7 HARMONY 2013: Connecticut . . . 8
2.2.8 HARMONY 2017: Seattle . . . 9
2.2.9 HARMONY 2018: Oxford . . . 9
2.2.10 HARMONY 2019: Pasadena . . . 9
**3 Proposed syntax and semantics** 10
3.1 Overview . . . 10
3.2 Namespace URI and other declarations necessary for using this package . . . 10
3.3 Primitive data types . . . 11
3.3.1 Type `ExternalRef` . . . 11
3.3.2 Type `UncertKind` . . . 11
3.4 Defining Distributions . . . 12
3.4.1 The approach . . . 12
3.5 Extended **Math** . . . 12
3.6 Discrete vs. continuous sampling . . . 13
3.7 Examples using the extended `csymbol` element . . . 14
3.7.1 Using a normal distribution . . . 14
3.7.2 Defining a truncated normal distribution . . . 15
3.7.3 Defining conditional events . . . 15
3.8 The **DistribBase** class . . . 17
3.9 The extended ***SBase*** class . . . 17
3.10 The **Uncertainty** class . . . 18
3.10.1 Attributes inherited from ***SBase*** . . . 19
3.11 The **UncertParameter** class . . . 19
3.11.1 The `type` attribute . . . 19
3.11.2 The `value` and `var` attributes . . . 20
3.11.3 The `units` attribute . . . 20
3.11.4 The `definitionURL` attribute . . . 21
3.11.5 Attributes inherited from ***SBase*** . . . 21
3.11.6 The child `math` element . . . 21
3.11.7 The child `ListOfUncertParameters` element . . . 21
3.12 The **UncertSpan** class . . . 21
3.13 The different **UncertParameter** and **UncertSpan** `type` values. . . . 21
3.14 The uncertainty of a **Species** . . . 25
3.15 Examples using **Uncertainty** . . . 26
3.15.1 Basic **Uncertainty** example . . . 26
3.15.2 Defining a random variable . . . 26
3.15.3 Defining external distributions . . . 27
**4 Interaction with other packages** 29
4.1 Custom annotations for function definitions . . . 29
4.2 The Arrays package . . . 30
4.3 SBML Level 3 Version 2 . . . 30
4.4 Other SBML Level 3 Packages . . . 31
**5 Use-cases and examples** 32
5.1 Sampling from a distribution: PK/PD Model . . . 32
5.2 Multiple uses of distributions . . . 34
5.3 Defining confidence intervals . . . 34
**A Validation of SBML documents** 37
A.1 Validation and consistency rules . . . 37

**B Acknowledgments** 43
**References** 44

## Revision History

The following table summarizes the history of this document.

| Version | Date | Author | Comments |
|---|---|---|---|
| 0.1 (Draft) | 15 Oct 2011 | Stuart Moodie | First draft |
| 0.2 (Draft) | 16 Oct 2011 | Stuart Moodie | Added introductory text and background info. Other minor changes etc. |
| 0.3 (Draft) | 16 Oct 2011 | Stuart Moodie | Filled empty invocation semantics section. |
| 0.4 (Draft) | 4 Jan 2012 | Stuart Moodie | Incorporated comments from Nicolas, Maciej and Sarah. Some minor revisions and corrections. |
| 0.5 (Draft) | 6 Jan 2012 | Stuart Moodie | Incorporated addition comments on aim of package from Nicolas. |
| 0.6 (Draft) | 19 Jul 2012 | Stuart Moodie | Incorporated revisions discussed and agreed at HARMONY 2012. |
| 0.7 (Draft) | 6 Aug 2012 | Stuart Moodie | Incorporated review comments from Maciej and Sarah. |
| 0.8 (Draft) | 21 Dec 2012 | Stuart Moodie | Incorporated changes suggested at combine and subsequently through list discussions. |
| 0.9 (Draft) | 9 Jan 2013 | Stuart Moodie | Incorporated corrections and comments from Maciej and Sarah. |
| 0.10 (Draft) | 10 Jan 2013 | Stuart Moodie | Modified based on comments from Maciej. |
| 0.11 (Draft) | 17 May 2013 | Lucian Smith | Modified based on Stuart's proposals and PWG discussion. |
| 0.12 (Draft) | June 2013 | Lucian Smith and Stuart Moodie | Modified based on HARMONY 2013 discussion. |
| 0.13 (Draft) | July 2013 | Lucian Smith and Stuart Moodie | Modified based PWG discussion, particularly with respect to UncertML. |
| 0.14 (Draft) | March 2015 | Lucian Smith | Modified to match UncertML 3.0. |
| 0.15 (Draft) | March 2015 | Lucian Smith and Sarah Keating | Modified to match UncertML 3.0 for real this time. |
| 0.16 (Draft) | March 2015 | Lucian Smith | Added information about UncertML 3.0 distributions, and the distributions custom annotations. |
| 0.17 (Draft) | June 2017 | Lucian Smith | Extensive update to reflect demise of UncertML 3.0, and appearance of ProbOnto. |
| 0.18 (Draft) | June 2017 | Lucian Smith | Fixes to reflect feedback on version 0.17. |
| 0.19 (Draft) | June 2018 | Lucian Smith | Resolved id/name issues with SBML Core L3V1 vs. L3V2. |
| 0.20 (Draft) | December 2018 | Lucian Smith | Updates to allow distributions as new MathML csymbols. |
| 0.21 (Draft) | January 2019 | Lucian Smith | Revisions based on suggestions from sbml-distrib, including extensive edits from Matthias. Extended function definitions removed. |
| 0.22 (Draft) | February 2019 | Lucian Smith | Addition of `sampleSize` and mean values of distributions for fallback. |
| 0.23 (Draft) | February 2019 | Lucian Smith and Michael Hucka | Removal of Distribution and all subclasses; replaced with a **Math** element instead; collapsed UncertStatistics into Uncertainty; some other edits. |
| 0.24 (Draft) | April 2019 | Lucian Smith | Removal of second distrib namespace, and consolidation of the **UncertParameter** class based on HARMONY 2019 discussions. |
| 0.25 (Draft) | July 2019 | Lucian Smith | The `id` of a **UncertParameter** no longer has mathematical meaning in any other context. |
| 0.26 (Release Candidate) | March 2020 | Lucian Smith | Add validation rules, and other corrections for release candidate. |
| 1.0 (Release) | April 2020 | Lucian Smith | Add qual example and adjust wording based on feedback from SBML Editors. |

## 1 Introduction and motivation

### 1.1 What is the Distributions package?

The Distributions package (also known as *distrib*) provides an extension to SBML Level 3 that extends MathML to allow draws from distributions, and also provides the ability to annotate model elements with information about the distribution their values were drawn from.

### 1.2 Scope

The Distributions package adds support to SBML for sampling from a probability distribution. In particular the following are in scope:

- Sampling from a univariate continuous distribution
- Sampling from a univariate discrete distribution
- Specification of descriptive statistics (mean, standard deviation, standard error, etc.)

At one point the following were considered for inclusion in this package but are now **out of scope**:

- Sampling from a multivariate distribution
- Definitions of ranges as new first-order objects (the 'Arrays' package now fills that objective)
- Sampling from user-defined probability density function
- Stochastic differential equations
- Other functions used to characterise a probability distribution, such as cumulative distribution functions (CDF) or survival functions, etc.

### 1.3 This document

This draft specification describes the consensus view of workshop participants and subscribers to the sbml-distrib mailing list. Although it was written by the listed authors, it does not solely reflect their views nor is it their proposal alone. Rather, it is their understanding of the consensus view of what the Distributions package should do and how it should do it. The contributors listed have made significant contributions to the development and writing of this specification and are credited accordingly, but a more comprehensive attribution is provided in the acknowledgments (Appendix B on page 43).

## 2 Background

### 2.1 Problems with current SBML approaches

SBML Level 3 Core has no direct support for encoding values sampled from distributions. Currently there is no workaround within the core SBML language itself, although it is possible to define the necessary information using annotations on SBML elements. Frank Bergmann proposed such an annotation scheme for use with SBML Levels 2 and 3 (see Section 4.1 on page 29).

### 2.2 Past work on this problem or similar topics

#### *2.2.1 Newcastle Proposal 2005*

In 2005, Colin Gillespie and others put forward a proposal [1] to introduce support for probability distributions in the SBML core specification. This was based on their need to use such distributions to represent the models they were creating as part of the BASIS project (`http://www.basis.ncl.ac.uk`). They proposed that distributions be referred to in SBML using the **csymbol** element in the MathML subset used by the SBML Core specification. An example is below:

```
<math xmlns=''http://www.w3.org/1998/Math/MathML''>
  <apply>
    <csymbol encoding=''text''
        definitionURL=''http://www.sbml.org/sbml/symbols/uniformRandom''>
      uniformRandom
    </csymbol>
    <ci>mu</ci>
    <ci>sigma</ci>
  </apply>
</math>
```

This required that a library of definitions be maintained as part of the SBML standard and in their proposal they defined an initial small set of commonly used distributions. The proposal was never implemented.

#### *2.2.2 SBML Hackathon 2010: Seattle*

The “distrib” package was discussed at the Seattle SBML Hackathon[2] and this section is an almost verbatim reproduction of Darren Wilkinson’s report on the meeting[3]. In the meeting, Darren presented an overview of the problem[45], building on the old proposal from the Newcastle group (see above: Section 2.2.1). There was broad support at the meeting for development of such a package, and for the proposed feature set. Discussion following the presentation led to consensus on the following points:

- There is an urgent need for such a package.
- It is important to make a distinction between a description of uncertainty regarding a model parameter and the mechanistic process of selecting a random number from a probability distribution, for applications such as parameter scans and experimental design
- It is probably worth including the definition of PMFs, PDFs and CDFs in the package
- It is worth including the definition of random distributions using particle representations within such a package, though some work still needs to be done on the precise representation

[1] `http://sbml.org/Community/Wiki/SBML_Leve\T1\l_3_Proposals/Distributions_and_Ranges`
[2] `http://sbml.org/Events/Hackathons/The_2010_SBML-BioModels.net_Hackathon`
[3] `http://sbml.org/Forums/index.php?t=tree&goto=6141&rid=0`
[4] Slides: `http://sbml.org/images/3/3b/Djw-sbml-hackathon-2010-05-04.pdf`
[5] Audio: `http://sbml.org/images/6/67/Wilkinson-distributions-2010-05-04.mov`

- It could be worth exploring the use of XML's `xinclude` construct to point at particle representations held in a separate file
- Random numbers must not be used in rate laws or anywhere else that is continuously evaluated, as then simulation behaviour is not defined
- Although there is a need for a package for describing extrinsic noise via stochastic differential equations in SBML, such mechanisms should not be included in this package due to the considerable implications for simulator developers
- We probably don't want to layer on top of UncertML (www.uncertml.org), as this spec is fairly heavy-weight, and somewhat tangential to our requirements
- A random number seed is not part of a model and should not be included in the package
- The definition of truncated distributions and the specification of hard upper and lower bounds on random quantities should be considered.

It was suggested that new constructs could be introduced into SBML via user-defined functions by embedding "distrib" constructs in a manner illustrated by the following example:

```
<listOfFunctionDefinitions>
  <functionDefinition id="myNormRand">
    <distrib:####>
      #### distrib binding information here ####
    </distrib:####>
    <math>
      <lambda>
        <bvar>
          <ci>mu</ci>
          <ci>sigma</ci>
        </bvar>
        <ci>mu</ci>
      </lambda>
    </math>
  </functionDefinition>
</listOfFunctionDefinitions>
```

This approach allows the use of a "default value" by simulators which do not understand the package (but simulators which do will ignore the <math> element). The package would nevertheless be "required", as it will not be simulated correctly by software which does not understand the package.

Informal discussions following the break-out covered topics such as:

- how to work with vector random quantities despite that SBML does not use the vector element from MathML
- how care must be taken with the semantics of random variables and the need to both:
  - reference multiple independent random quantities at a given time
  - make multiple references to the same random quantity at a given time

#### 2.2.3 Statistical Models Workshop 2011: Hinxton

Detailed discussion was continued at the Statistical Models Workshop in Hinxton in June 2011[6]. There, people interested in representing statistical models in SBML came together to work out the details of how this package would work in detail. Dan Cornford from the UncertML project[7] attended the meeting and described how UncertML could be used to describe uncertainty and in particular probability distributions. Perhaps the most significant

[6] http://sbml.org/Events/Other_Events/statistical_models_workshop_2011
[7] http://www.uncertml.org/

decision at this meeting was to adopt the UncertML resource as a controlled vocabulary that is referenced by the Distributions package.

Much has changed since this meeting, but the output from this meeting was the basis for the first version of the "distrib" draft specification.

#### 2.2.4 HARMONY 2012: Maastricht

Two sessions were dedicated to discussion of Distributions at HARMONY based around the proposals described in version 0.5 of this document. In addition there was discussion about the Arrays proposal which was very helpful in solving the problem of multivariate distributions in Distributions. The following were the agreed outcomes of the meeting:

- The original "distrib" draft included UncertML markup directly in the function definition. This proved unwieldy and confusing and has been replaced by a more elegant solution that eliminates the UncertML markup and integrates well with the fallback function (see details below).
- Multivariate distributions can be supported using the Arrays package to define a covariance matrix.
- User defined continuous distributions would define a PDF in MathML.
- Usage semantics were clarified so that invokation of a function definition implied a value was sampled from the specified distribution.
- It was agreed from which sections of an SBML model a distribution could be invoked.
- Statistical descriptors of variables (for example mean and standard deviation) would be separated from Distributions and either provided in a new package or in a later version of SBML L3 core.

#### 2.2.5 COMBINE 2012: Toronto

The August draft of "distrib" was reviewed, and an improvement was agreed upon in the user-defined PMF part of the proposal. In particular, is was agreed that the categories should be defined by *distrib* classes rather than by passing in the information as an array. Questions were also raised about whether UncertML was suitably well defined to be used as an external definition for probability distributions. This was resolved subsequent to the meeting with a teleconference to Dan Cornford and colleagues. These changes are incorporated here. Finally, there was considerable debate about whether to keep the dependence of *distrib* on the Arrays package in order to support multi-variate distributions. The outcome was an agreement that we would review this at the end of 2012, based on the results of an investigation into how feasible it would be to implement Arrays as a package.

#### 2.2.6 Package Working Group discussions 2013

Early 2013 saw a good amount of discussion on the *distrib* Package Working Group mailing list, spurred by proposals by Stuart Moodie[8]. While not all of his suggestions ended up being fully accepted by the group, several changes were accepted, including:

- To use UncertML as actual XML, instead of as a set of reference definitions.
- To use UncertML to encode descriptive statistics of SBML elements such as mean, standard deviation, standard error, etc.) bringing this capability back in scope for this package.

#### 2.2.7 HARMONY 2013: Connecticut

At the HARMONY held at the University of Connecticut Health Center, further discussions revealed the importance of distinguishing the ability to describe an element as a distributed variable vs. a function call within the model performing a draw from a distribution.

[8] http://thestupott.wordpress.com/2013/03/12/an-improved-distrib-proposal/

We also decided to discard the encoding of explicit PDFs for now, as support for it is remarkably complicated, and there no demand for it. The current design could be extended to support this feature so if there is demand for it in the future support for explicit PDFs could be reintroduced.

#### 2.2.8 HARMONY 2017: Seattle

In early 2017, it became clear that UncertML was no longer being worked on; the web page had lapsed, and its authors had moved on to other things. At the same time, the ProbOnto ontology (Swat et al. 2016; `http://probonto.org/`) was developed that included all the distributions from UncertML as well as a huge number of other distributions. On the "distrib" mailing list, there was discussion about whether to create essentially our own version of UncertML, or to implement a generic "reference" format that used ProbOnto. The v0.17 draft specification was developed as a compromise 'hybrid' system that did parts of both, so that basic distributions would be hard-coded, but the ability to reference any ProbOnto ontology would also be present. The hope is that with working examples of both approaches, either the hybrid approach will be approved, or if one is preferred, the other approach may be removed. This version of the specification was created for presentation at HARMONY 2017 in Seattle.

#### 2.2.9 HARMONY 2018: Oxford

At the HARMONY held at the University of Oxford, for the first time since the change from UncertML, a libSBML implementation of the specification was available. This let people experiment with the package, and conclude that a simpler method of defining calls to distributions was desired. It was proposed to define new MathML `csymbol` definitions for the common distributions. Eventually, these new `csymbols` were used instead of the old Distribution class, greatly simplifying the proposal.

#### 2.2.10 HARMONY 2019: Pasadena

In the weeks leading up to the HARMONY held at Caltech, the PWG discussed various options for encoding uncertainties, based on different people's requirements. At HARMONY, we were able to coalesce around an approach that seems likely to work for everyone, with multiple uncertainties per element, and a single **UncertParameter** class with a `type` that encapsulates what used to be encoded in an element's class.

## 3 Proposed syntax and semantics

### 3.1 Overview

We use UML 1.0 (Unified Modeling Language; Eriksson and Penker 1998; Oestereich 1999) class diagram notation to define the constructs provided by this package. We also use color in the diagrams to provide additional information for the benefit of those viewing the document on media that can display color. The following are the colors used and what they represent:

- *Black*: Items colored black in the UML diagrams are components taken unchanged from their definition in the SBML Level 3 Core specification document.
- *Green*: Items colored green are components that exist in SBML Level 3 Core, but are extended by this package. Class boxes are also drawn with dashed lines to further distinguish them.
- *Blue*: Items colored blue are new components introduced in this package specification. They have no equivalent in the SBML Level 3 Core specification.
- *Red lines*: Classes with red lines in the corner are fully defined in a different figure.

We also use the following typographical conventions to distinguish the names of objects and data types from other entities; these conventions are identical to the conventions used in the SBML Level 3 Core specification document:

***AbstractClass***: Abstract classes are never instantiated directly, but rather serve as parents of other classes. Their names begin with a capital letter and they are printed in a slanted, bold, sans-serif typeface. In electronic document formats, the class names defined within this document are also hyperlinked to their definitions; clicking on these items will, given appropriate software, switch the view to the section in this document containing the definition of that class. (However, for classes that are unchanged from their definitions in SBML Level 3 Core, the class names are not hyperlinked because they are not defined within this document.)

**Class**: Names of ordinary (concrete) classes begin with a capital letter and are printed in an upright, bold, sans-serif typeface. In electronic document formats, the class names are also hyperlinked to their definitions in this specification document. (However, as in the previous case, class names are not hyperlinked if they are for classes that are unchanged from their definitions in the SBML Level 3 Core specification.)

`SomeThing`, `otherThing`: Attributes of classes, data type names, literal XML, and tokens *other* than SBML class names, are printed in an upright typewriter typeface. Primitive types defined by SBML begin with a capital letter; SBML also makes use of primitive types defined by XML Schema 1.0 (Biron and Malhotra, 2000; Fallside, 2000; Thompson et al., 2000), but unfortunately, XML Schema does not follow any capitalization convention and primitive types drawn from the XML Schema language may or may not start with a capital letter.

`[elementName]`: In some cases, an element may contain a child of any class inheriting from an abstract base class. In this case, the name of the element is indicated by giving the abstract base class name in brackets, meaning that the actual name of the element depends on whichever subclass is used. The capitalization follows the capitalization of the name in brackets.

For other matters involving the use of UML and XML, we follow the conventions used in the SBML Level 3 Core specification document.

### 3.2 Namespace URI and other declarations necessary for using this package

Every SBML Level 3 package is identified uniquely by an XML namespace URI. For an SBML document to be able to use a given Level 3 package, it must declare the use of that package by referencing its URI. This version of the Distributions package uses the URI:

“`http://www.sbml.org/sbml/level3/version1/distrib/version1`”

Note that the Distributions package may be used with both SBML Level 3 Version 1 and SBML Level 3 Version 2 documents, with no semantic changes between the two in any *distrib* element, due to the addition of `id` and `name` to the **DistribBase** class.

In addition, SBML documents using a given package must indicate whether the package may be used to change the mathematical meaning of SBML Level 3 Core elements. This is done using the attribute `required` on the `<sbml>` element in the SBML document. For the Distributions package, the value of this attribute must be "`true`", as it defines new csymbols that may be used in any MathML. Note that the value of this attribute must *always* be set to "`true`", even if the particular model does not contain any of these csymbols.

The following fragment illustrates the beginning of a typical SBML model using SBML Level 3 Version 1 and this version of the Distributions package:

```
<?xml version="1.0" encoding="UTF-8"?>
<sbml xmlns="http://www.sbml.org/sbml/level3/version1/core" level="3" version="1"
      xmlns:distrib="http://www.sbml.org/sbml/level3/version1/distrib/version1"
      distrib:required="true">
```

The following fragment illustrates the beginning of a typical SBML model using SBML Level 3 Version 2 and this version of the Distributions package:

```
<?xml version="1.0" encoding="UTF-8"?>
<sbml xmlns="http://www.sbml.org/sbml/level3/version2/core" level="3" version="2"
      xmlns:distrib="http://www.sbml.org/sbml/level3/version1/distrib/version1"
      distrib:required="true">
```

There is no difference between the 'distrib' part of these documents, and all package semantics are identical.

##### XML Namespace use

For element names, XML has clear rules about how to declare and use namespaces. In typical SBML documents, the Distributions namespace will be defined as above, and elements will therefore need to be prefixed with "`distrib:`".

In contrast to element names, XML *attribute* names are completely defined by the element in which they appear, and never have a "default" namespace defined. The element itself declares whether any attributes should be defined with a namespace prefix.

Following the typical convention used by SBML packages, any attribute that appears in a UML diagram in this specification may *either* be defined with no namespace prefix, *or* be defined with the *distrib* namespace as a prefix. (No attributes are defined here as extentions of existing core SBML elements, and thus none of them are required to have the *distrib* namespace as a prefix.)

### 3.3 Primitive data types

The Distributions package uses data types described in Section 3.1 of the SBML Level 3 Core specification, and adds the additional primitive types described below.

#### 3.3.1 Type `ExternalRef`

The type `ExternalRef` is derived from the type `string` with the additional requirement that it be a valid URI. An `ExternalRef` is used in the **UncertParameter** class to point to ontologies such as ProbOnto (Swat et al., 2016), which contain the definitions of distributions and parameters.

#### 3.3.2 Type `UncertKind`

The type `UncertKind` is derived from the type `string` and its values are restricted to being one of the following possibilities: "`coefficientOfVariation`", "`kurtosis`", "`mean`", "`median`", "`mode`", "`sampleSize`", "`skewness`",

"standardDeviation", "standardError", "variance", "confidenceInterval", "credibleInterval", "interquartileRange", "range", "externalParameter", and "distribution". Attributes of type UncertKind cannot take on any other values. The meaning of these values is discussed in the context of the **UncertParameter** class's definition in Section 3.11 on page 19.

### 3.4 Defining Distributions

#### 3.4.1 The approach

The Distributions package has two simple purposes. First, it provides a mechanism for sampling a random value from a probability distribution. This implies that it must define the probability distribution and then must sample a random value from that distribution. Second, it provides a mechanism for describing elements with information about their uncertainty. An example use case for this is to provide the standard deviation for a value. Another might be describing a parameter's distribution so it could be used in a parameter scan experiment.

Sampling from probability distributions is achieved by allowing new MathML elements, and encoding uncertainty by extending ***SBase***, which in turn uses the **Uncertainty** class. Several distributions and statistics are defined explicitly in this specification, but more can be defined by referencing an external ontology such as ProbOnto through the **UncertParameter** class.

When a call to a distribution is defined in the extended **Math**, it is sampled when it is invoked. If a particular sampled value should be used multiple times, that value must be assigned to a parameter first, such as through the use of an **InitialAssignment** or **EventAssignment**. When an external distribution is defined, it is not used in the math of the model, but may be used externally where appropriate.

### 3.5 Extended Math

To allow quick access to a variety of common functions, the Distributions package allows the use of new types of csymbol elements anywhere that **Math** is used. These csymbols are functions, and therefore must be the first child of an apply element, and their arguments are predefined: you cannot call *normal(mean, variance)*, because the definition of the normal csymbol is *normal(mean, stdev)*.

The newly-allowed csymbol elements are defined in Table 1 on the next page.

Many of the distributions take exactly two or four arguments (or exactly one or three arguments). For those functions, the optional last two arguments are *min* and *max*, for when the draw from the distribution is constrained to be between those two values. For all functions, the *min* boundary is inclusive; that is, a value of *min* may be returned by the function (though this may be very unlikely for draws from a continuous distribution). For all continuous distributions, the *max* boundary is *not* inclusive; that is, a value of *max* will never be returned. The continuous distributions are normal, cauchy, chisquare, exponential, gamma, laplace, lognormal, and rayleigh. For the discrete distributions, the *max* boundary is inclusive: that is, a value of *max* may indeed be returned. The discrete distributions are binomial and poisson.

The value of min must be less than the value of max for all continuous distributions, and the value of min must be less than or equal to the value of max for all discrete distributions. Additionally, the min and max values of a discrete distribution must span at least one integer between them, inclusive.

To define a distribution with only one bound, the other bound should be defined as INF or -INF, as appropriate. For those distributions that have an intrisic lower bound of 0, setting min to 0 or any negative number will have no effect, but is legal.

The versions of cauchy and laplace with one argument draw from the corresponding distribution with that argument as its scale value, and a value of "0" for its location.

***Table 1:** The "`definitionURL`" values allowed for the `csymbol` of **Math** for documents that use the distrib package, and the arguments those functions may take.*

| URI | Possible arguments |
|---|---|
| `http://www.sbml.org/sbml/symbols/distrib/normal` | *normal(mean, stdev)*<br>*normal(mean, stdev, min, max)* |
| `http://www.sbml.org/sbml/symbols/distrib/uniform` | *uniform(min, max)* |
| `http://www.sbml.org/sbml/symbols/distrib/bernoulli` | *bernoulli(prob)* |
| `http://www.sbml.org/sbml/symbols/distrib/binomial` | *binomial(nTrials, probabilityOfSuccess)*<br>*binomial(nTrials, probabilityOfSuccess, min, max)* |
| `http://www.sbml.org/sbml/symbols/distrib/cauchy` | *cauchy(scale)*<br>*cauchy(location, scale)*<br>*cauchy(location, scale, min, max)* |
| `http://www.sbml.org/sbml/symbols/distrib/chisquare` | *chisquare(degreesOfFreedom)*<br>*chisquare(degreesOfFreedom, min, max)* |
| `http://www.sbml.org/sbml/symbols/distrib/exponential` | *exponential(rate)*<br>*exponential(rate, min, max)* |
| `http://www.sbml.org/sbml/symbols/distrib/gamma` | *gamma(shape, scale)*<br>*gamma(shape, scale, min, max)* |
| `http://www.sbml.org/sbml/symbols/distrib/laplace` | *laplace(scale)*<br>*laplace(location, scale)*<br>*laplace(location, scale, min, max)* |
| `http://www.sbml.org/sbml/symbols/distrib/lognormal` | *lognormal(mean, stdev)*<br>*lognormal(mean, stdev, min, max)* |
| `http://www.sbml.org/sbml/symbols/distrib/poisson` | *poisson(rate)*<br>*poisson(rate, min, max)* |
| `http://www.sbml.org/sbml/symbols/distrib/rayleigh` | *rayleigh(scale)*<br>*rayleigh(scale, min, max)* |

##### *Fallback functions*

If an SBML interpreter is unable to calculate one or more of the above extended MathML functions, it may simply fail, or it might choose to return the mean of the given function instead. In either case, it is a good idea to inform the user that the model cannot be interpreted by the software as intended. Note that the mean of a discrete distribution is not necessarily a legal return value for that function, as it may not be an integer.

The mean values in Table 2 on the following page may be used as a fallback for software that cannot perform draws from a distribution. Note that truncated versions of these functions will have different means. Note also that the `cauchy` distribution has no mean, by definition.

### 3.6 Discrete vs. continuous sampling

MathML csymbols may be used in SBML Level 3 Core in both discrete and continuous contexts: **InitialAssignment**, **EventAssignment**, **Priority**, and **Delay** elements are all discrete, while **Rule**, **KineticLaw**, and **Trigger** elements are all continuous in time. For discrete contexts, the behavior of *distrib*-extended **FunctionDefinition** elements is well-defined: a single random value is sampled from the distribution each time the function definition is invoked. Each invocation implies one sampling operation. In continuous contexts, however, their behavior is ill-defined. More information than is defined in this package (such as autocorrelation values or full conditional probabilities) would

***Table 2:*** *The mean values for the non-truncated versions of the distribution functions. These values could potentially be used as a fallback for simulators which are not able to draw from the distributions themselves.*

| Function | Fallback (mean) |
|---:|---|
| *normal(mean, stdev)* | *mean* |
| *uniform(min, max)* | $\frac{min+max}{2}$ |
| *bernoulli(prob)* | *prob* |
| *binomial(nTrials, probabilityOfSuccess)* | *nTrials* × *probabilityOfSuccess* |
| *cauchy(location, scale)* | undefined |
| *chisquare(degreesOfFreedom)* | *degreesOfFreedom* |
| *exponential(rate)* | $rate^{-1}$ |
| *gamma(shape, scale)* | *shape* × *scale* |
| *laplace(location, scale)* | *location* |
| *lognormal(mean, stdev)* | $exp(mean + stdev^2/2)$ |
| *poisson(rate)* | *rate* |
| *rayleigh(scale)* | $scale\sqrt{\pi/2}$ |

be required to make random sampling tractable in continuous contexts, and is beyond the scope of this version of the package. If some package is defined in the future that adds this information, or if custom annotations are provided that add this information, such models may become simulatable. However, this package does not define how to handle sampling in continuous contexts, and recommends against it: a warning may be produced by any software encountering the use of a *distrib*-extended MathML in a continuous context. Assuming such models are desirable, and the information is not provided in a separate package, this information may be incorporated into a future version of this specification.

Any other package that defines new contexts for MathML will also be either discrete or continuous. Discrete situations (such as those defined in the SBML Level 3 Qualitative Models package) are, as above, well-defined. Continuous situations (as might arise within the Spatial Processes package, over space instead of over time) will most likely be ill-defined. Those packages must therefore either define for themselves how to handle *distrib*-extended MathML elements, or leave it to some other package/annotation scheme to define how to handle the situation.

### 3.7 Examples using the extended `csymbol` element

Several examples are given below that illustrate various uses of the new `csymbol` elements introduced by *distrib*.

#### *3.7.1 Using a normal distribution*

In this example, the initial value of `y` is set as a draw from the normal distribution $normal(z, 10)$:

```
<initialAssignment symbol="y">
  <math xmlns="http://www.w3.org/1998/Math/MathML">
    <apply>
      <csymbol definitionURL="http://www.sbml.org/sbml/symbols/distrib/normal"
               encoding="text"> normal </csymbol>
      <ci> z </ci>
      <cn> 10 </cn>
    </apply>
  </math>
</initialAssignment>
```

This use would apply a draw from a normal distribution with mean `z` and standard deviation `10` to the symbol `y`.

#### 3.7.2 Defining a truncated normal distribution

When used with four arguments instead of two, the normal distribution is truncated to $normal(z, 10, z-2, z+2)$:

```
<initialAssignment symbol="y">
  <math xmlns="http://www.w3.org/1998/Math/MathML">
    <apply>
      <csymbol definitionURL="http://www.sbml.org/sbml/symbols/distrib/normal"
               encoding="text"> normal </csymbol>
      <ci> z </ci>
      <cn type="integer"> 10 </cn>
      <apply>
        <minus/>
        <ci> z </ci>
        <cn type="integer"> 2 </cn>
      </apply>
      <apply>
        <plus/>
        <ci> z </ci>
        <cn type="integer"> 2 </cn>
      </apply>
    </apply>
  </math>
</initialAssignment>
```

This use would apply a draw from a normal distribution with mean `z`, standard deviation `10`, lower bound `z - 2` (inclusive) and upper bound `z + 2` (not inclusive) to the SBML symbol `y`.

#### 3.7.3 Defining conditional events

Simultaneous events in SBML are ordered based on their **Priority** values, with higher values being executed first, and potentially cancelling events that fire after them. In this example, two simultaneous events have priorities set with csymbols defined in *distrib*. The event `E0` has a priority of $uniform(0,1)$, while the event `E1` has a priority of $uniform(0,2)$. This means that 75% of the time, event `E1` will have a higher priority than `E0`, and will fire first, assigning a value of `5` to parameter `x`. Because this negates the trigger condition for `E0`, which is set `persistent`="`false`", this means that `E0` never fires, and the value of `x` remains at `5`. The remaining 25% of the time, the reverse happens, with `E0` setting the value of `x` to `3` instead.

```
<?xml version="1.0" encoding="UTF-8"?>
<sbml xmlns="http://www.sbml.org/sbml/level3/version2/core"
      xmlns:distrib="http://www.sbml.org/sbml/level3/version1/distrib/version1"
      level="3" version="2" distrib:required="true">
  <model metaid="__main" id="__main">
    <listOfParameters>
      <parameter metaid="__main.x" id="x" value="0" constant="false"/>
    </listOfParameters>
    <listOfEvents>
      <event id="E0" useValuesFromTriggerTime="true">
        <trigger initialValue="true" persistent="false">
          <math xmlns="http://www.w3.org/1998/Math/MathML">
            <apply>
              <and/>
              <apply>
                <gt/>
                <csymbol encoding="text" definitionURL="http://www.sbml.org/sbml/symbols/time">
                         time </csymbol>
                <cn type="integer"> 2 </cn>
              </apply>
              <apply>
                <lt/>
```

```
                <ci> x </ci>
                <cn type="integer"> 1 </cn>
              </apply>
            </apply>
          </math>
        </trigger>
        <priority>
          <math xmlns="http://www.w3.org/1998/Math/MathML">
            <apply>
              <csymbol definitionURL="http://www.sbml.org/sbml/symbols/distrib/uniform"
                       encoding="text"> uniform  </csymbol>
              <cn type="integer"> 0 </cn>
              <cn type="integer"> 1 </cn>
            </apply>
          </math>
        </priority>
        <listOfEventAssignments>
          <eventAssignment variable="x">
            <math xmlns="http://www.w3.org/1998/Math/MathML">
              <cn type="integer"> 3 </cn>
            </math>
          </eventAssignment>
        </listOfEventAssignments>
      </event>
      <event id="E1" useValuesFromTriggerTime="true">
        <trigger initialValue="true" persistent="false">
          <math xmlns="http://www.w3.org/1998/Math/MathML">
            <apply>
              <and/>
              <apply>
                <gt/>
                <csymbol encoding="text" definitionURL="http://www.sbml.org/sbml/symbols/time">
                         time </csymbol>
                <cn type="integer"> 2 </cn>
              </apply>
              <apply>
                <lt/>
                <ci> x </ci>
                <cn type="integer"> 1 </cn>
              </apply>
            </apply>
          </math>
        </trigger>
        <priority>
          <math xmlns="http://www.w3.org/1998/Math/MathML">
            <apply>
              <csymbol definitionURL="http://www.sbml.org/sbml/symbols/distrib/uniform"
                       encoding="text"> uniform  </csymbol>
              <cn type="integer"> 0 </cn>
              <cn type="integer"> 2 </cn>
            </apply>
          </math>
        </priority>
        <listOfEventAssignments>
          <eventAssignment variable="x">
            <math xmlns="http://www.w3.org/1998/Math/MathML">
              <cn type="integer"> 5 </cn>
            </math>
          </eventAssignment>
        </listOfEventAssignments>
      </event>
    </listOfEvents>
  </model>
</sbml>
```

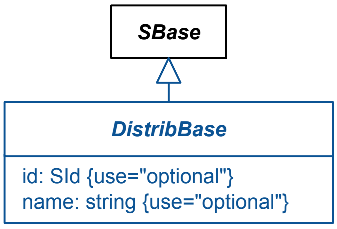

***Figure 1:*** *The definition of the **DistribBase** class. The* `id` *and* `name` *attributes defined are optional, and are identical to the ones they inherit in SBML Level 3 Version 2 documents from **SBase**.*

### 3.8 The DistribBase class

The **DistribBase** class is an abstract base class which is the parent class for every class in this Distributions package. Its purpose is to replicate within the Distributions package an important change between SBML Level 3 Version 1 and SBML Level 3 Version 2: the addition of an optional `id` and `name` attribute to ***SBase***. By adding these attributes here, *distrib* may be used completely exchangeably between Level 3 Version 1 and Level 3 Version 2 documents without any other modifications. The meaning of these attributes is identical, regardless of the Level/Version of the document in which they appear.

The `id` attribute is of type `SId`, and must be unique among other ids in the `SId` namespace in the parent **Model**, and has no mathematical meaning, unless stated otherwise in the definition of that object. The `name` attribute is of type `string`, and is provided to allow the user to define a human-readable label for the object. It has no uniqueness restrictions.

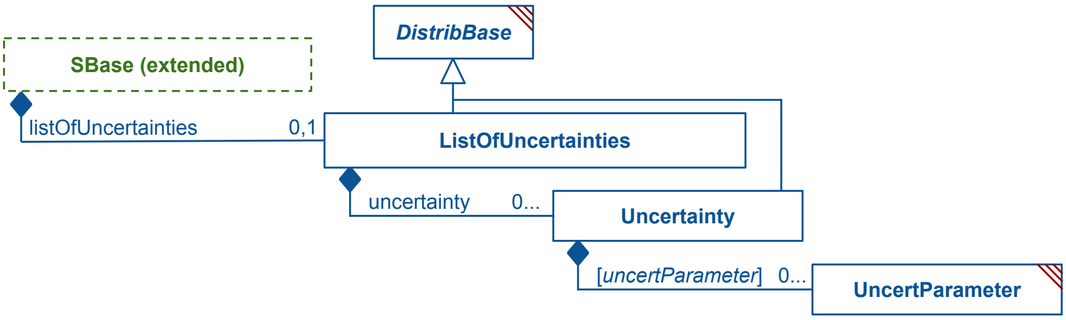

***Figure 2:*** *The definition of the extended **SBase** class to include a new optional **ListOfUncertainties** child element. Intended for use with any element with mathematical meaning, or with a **Math** child element. Also defines the **ListOfUncertainties** and **Uncertainty** classes.*

### 3.9 The extended *SBase* class

As can be seen in Figure 2, the SBML base class ***SBase*** is extended to include an optional **ListOfUncertainties** child element, which in turn contains optional **Uncertainty** elements, each of which may contain a set of **UncertParameter** objects that describe the uncertainty of the extended element. Multiple **Uncertainty** elements are allowed as children of ***SBase*** to allow the modeler to record **Uncertainty** measurements from different sources (papers, experiments, etc.) that may overlap and/or contradict one another.

In SBML Level 3 Core, one should only extend those ***SBase*** elements with mathematical meaning (**Compartment**, **Parameter**, **Reaction**, **Species**, and **SpeciesReference**), or those ***SBase*** elements with **Math** children (**Constraint**, **Delay**, **EventAssignment**, **FunctionDefinition**, **InitialAssignment**, **KineticLaw**, **Priority**, **Rule**, and **Trigger**). The **Uncer-**

**tainty** child is added to ***SBase*** instead of to each SBML element so that other packages inherit the ability to extend their own elements in the same fashion: for example, the Qualitative Models package has the **QualitativeSpecies** class which has mathematical meaning, and a **FunctionTerm** class which has a **Math** child. Both could be given an **Uncertainty** child containing information about the distribution or set of samples from which they were drawn.

A few SBML elements can interact in interesting ways that can confuse the semantics here. A **Reaction** element and its **KineticLaw** child, for example, both reference the same mathematical formula, so only one should be extended with an **Uncertainty** child element. Similarly, the uncertainty of an **InitialAssignment** will be identical to the uncertainty of the element it assigns to, and therefore only one of those elements should be extended.

Other elements not listed above should probably not be given an **Uncertainty** child, as it would normally not make sense to talk about the uncertainty of something that doesn't have a corresponding mathematical meaning. However, because packages or annotations can theoretically give new meaning (including mathematical meaning) to elements that previously did not have them, this is not a requirement.

It is important to note that the uncertainty described is defined as being the uncertainty at the moment the element's mathematical meaning is calculated, and does not describe the uncertainty of how that element changes over time. For a **Species**, **Parameter**, **Compartment**, and **SpeciesReference**, this means that it is the uncertainty of their initial values, and does not describe the uncertainty in how those values evolve in time. The reason for this is that other SBML constructs all describe how (or if) the values change in time, and it is those other constructs that should be used to describe a symbol's time-based uncertainty. For example, a **Species** whose initial value had uncertainty due to instrument precision could have an **Uncertainty** child describing this. A **Species** whose value was known to change over time due to unknown processes, but which had a known average and standard deviation could be given an **AssignmentRule** that set that **Species** amount to the known average, and the **AssignmentRule** itself could be given an **Uncertainty** child describing the standard deviation of the variability.

### 3.10 The Uncertainty class

The **Uncertainty** class is a collection of zero or more statistical measures related to the uncertainty of the parent SBML element. It may only contain one of each type of measurement, which means that each of its **UncertParameter** children must have a unique `type` attribute for every value but "`externalParameter`". Each **UncertParameter** child with a `type` of "`externalParameter`" must, in turn, have a unique `definitionURL` value. If a given SBML element has multiple measures of the same `type` (for example, as measured from different sources or different experiments), it should be given multiple **Uncertainty** children. Each **Uncertainty** child must be a unique set of statistical measures.

These statistical measures do not numerically affect simulation of the model. They are, in essence, a controlled annotation format specifically designed for this sort of information. Tools may use this information as they wish, just as they can with other annotation information.

Note that for elements that change in value over time, the described uncertainty applies only to the element's initial state, and not to how it changes in time. For typical simulations, this means the element's initial assignment.

The child **UncertParameter** children are named according to their class, so any **UncertSpan** child will have the element name `uncertSpan`, and any **UncertParameter** base class child will have the element name `uncertParameter`.

#### *Propagation of error*

It may be possible to propagate the error defined in **Uncertainty** elements through the mathematics defined in a simulation of the model. Be advised that this will be a complicated system, and may involve calculating partial derivates of equations that are not explicitly encoded. Many simulators choose instead to estimate the error through stochastic simulations. Either approach should be possible with a properly encoded *distrib* model.

#### 3.10.1 Attributes inherited from SBase

An **Uncertainty** always inherits the optional `metaid` and `sboTerm` attributes, and inherits optional `id` and `name` attributes as described in Section 3.8 on page 17. The `id` of an **Uncertainty** has no mathematical meaning.

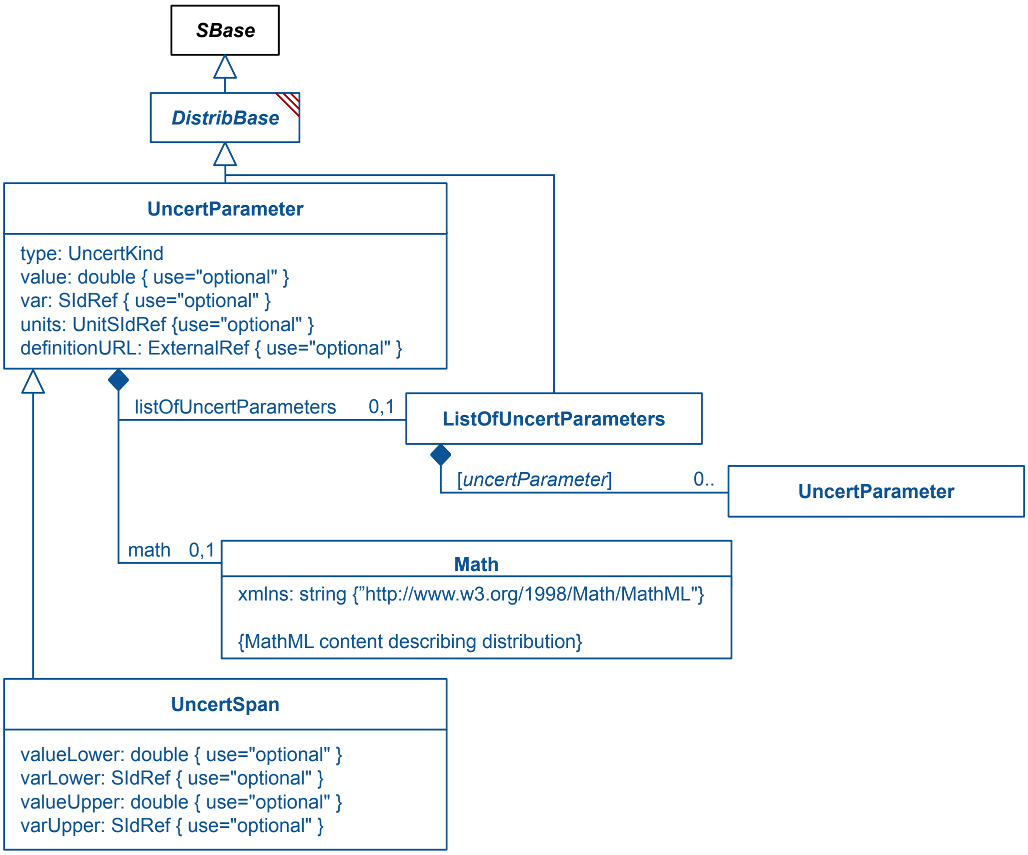

*__Figure 3:__ The definition of the __UncertParameter__, __UncertSpan__, and __ListOfUncertParameters__ classes. These classes allow an __Uncertainty__ to define an uncertainty numerically.*

### 3.11 The UncertParameter class

Each **UncertParameter** defines one uncertainty statistic about the parent element. It has one required attribute `type` of type `UncertKind` which defines what statistic it describes (i.e. "`mean`", "`standardDeviation`", "`distribution`", etc.). Its other attributes (`value`, `var`, `units`, and `definitionURL`), and children (`math` and `listOfUncertParameters`) are all optional, each useable according to which `type` it is.

#### 3.11.1 The `type` attribute

The `type` attribute defines what the **UncertParameter** describes. Depending on the type, other attributes will be allowed or not, and the class must either be the base **UncertParameter** or the **UncertSpan** class, according to Table 3 on the next page.

*Table 3: Values for the* `type` *attribute of a* ***UncertParameter****, the class that should be used with that type, and the attributes and children that are allowed.*

| Value | Class | units | value/var | valueLower/ varLower | valueUpper/ varUpper | definitionURL | math | listOfUncertParameters |
|---|---|---|---|---|---|---|---|---|
| *coefficientOfVariation* | **UncertParameter** | ✓ | ✓ | | | | | |
| *kurtosis* | **UncertParameter** | ✓ | ✓ | | | | | |
| *mean* | **UncertParameter** | ✓ | ✓ | | | | | |
| *median* | **UncertParameter** | ✓ | ✓ | | | | | |
| *mode* | **UncertParameter** | ✓ | ✓ | | | | | |
| *sampleSize* | **UncertParameter** | ✓ | ✓ | | | | | |
| *skewness* | **UncertParameter** | ✓ | ✓ | | | | | |
| *standardDeviation* | **UncertParameter** | ✓ | ✓ | | | | | |
| *standardError* | **UncertParameter** | ✓ | ✓ | | | | | |
| *variance* | **UncertParameter** | ✓ | ✓ | | | | | |
| *confidenceInterval* | **UncertSpan** | ✓ | | ✓ | ✓ | | | |
| *credibleInterval* | **UncertSpan** | ✓ | | ✓ | ✓ | | | |
| *interquartileRange* | **UncertSpan** | ✓ | | ✓ | ✓ | | | |
| *range* | **UncertSpan** | ✓ | | ✓ | ✓ | | | |
| *distribution* | **UncertParameter** | ✓ | | | | ✓ | ✓ | ✓ |
| *externalParameter* | *either* | ✓ | ✓ | ✓ | ✓ | ✓ | ✓ | ✓ |

#### *3.11.2 The `value` and `var` attributes*

The optional `value` attribute (of type `double`) is used when the **UncertParameter** equals the given number, and the optional `var` attribute (of type `SIdRef`) is used when the value of an **UncertParameter** equals the referenced element with mathematical meaning. Either attribute may be used for those **UncertParameter** types with a single value, but not both.

#### *3.11.3 The `units` attribute*

The optional `units` attribue of an **UncertParameter** is of type `UnitSIdRef`. The `UnitSIdRef` is defined in the SBML Level 3 Core specification, but in brief, it may either be the `SId` of a **UnitDefinition** in the **Model**, or a predefined SI unit from the Table 2 in the SBML Level 3 Core specification. The units of uncertainty statistics are generally either dimensionless or the same as the units of the parent, according to the formula that defines the value. A `mean` and a `standardDeviation`, for example, are always the same units as the parent, while a `coefficientOfVariation` is dimensionless.

#### 3.11.4 The definitionURL attribute

The optional definitionURL attribute (of type ExternalRef) may be used when the type of the UncertParameter is "distribution", and must be used when the type of the UncertParameter is "externalParameter". The ExternalRef should point to an ontology URL, distribution csymbol, or other unique definition string that defines what is meant by this UncertParameter. The definitionURL must not be defined if the type is any other value: the other types are already completely defined.

#### 3.11.5 Attributes inherited from SBase

An UncertParameter always inherits the optional metaid and sboTerm attributes, and inherits optional id and name attributes as described in Section 3.8 on page 17. The id of a UncertParameter does not take on the mathematical value of its value attribute, and may not be used in mathematical contexts. Instead, if the value of the element is to be used elsewhere, the var attribute should be used instead, and that referenced value used in other contexts.

#### 3.11.6 The child math element

The optional math element contains MathML, and may only be used for an UncertParameter of type "distribution" or "externalParameter". When defined for a "distribution", the MathML should define that distribution, such as by using one of the extended csymbol definitions from this specification.

#### 3.11.7 The child ListOfUncertParameters element

The optional child ListOfUncertParameters element may only be used for an UncertParameter of type "distribution" or "externalParameter". Unlike an Uncertainty, there are no uniqueness restrictions among the children of this element: any number of UncertParameter elements of any type may be used, according to whatever makes sense for the statistic defined by the parent definitionURL.

### 3.12 The UncertSpan class

The UncertSpan class defines a span of values that define an uncertainty statistic such as confidence interval or range. It inherits from UncertParameter, and adds four optional attributes, varLower and varUpper, of type SIdRef, and valueLower and valueUpper, of type double. Exactly one of the attributes varLower and valueLower may be defined, and exactly one of the attributes varUpper and valueUpper may be defined. If no attributes are defined, the parameters of the span are undefined. If only one attribute is defined (one of the upper or lower attributes), that aspect of the span is defined, and the other end is undefined. The span is fully defined if two attributes (one lower and one upper) are defined.

The value of the lower attribute (whichever is defined) must be lesser or equal to the value of the upper attribute (whichever is defined), at the initial conditions of the model. The Uncertainty element cannot affect the core mathematics of an SBML model, but if it is used in a mathematical context during simulation of the model, this restriction on the attribute values must be maintained, or the UncertSpan object as a whole will be undefined.

Like the units attribute on an UncertParameter, the units attribute is provided if valueUpper and/or valueLower is defined. The units on both the upper and lower ends of the span must match each other, if defined. The units for span ends defined by reference may be obtained from the referenced SBML element.

### 3.13 The different UncertParameter and UncertSpan type values.

The UncertKind values each have a particular definition. The following kinds are all single-value types, and thus may either be defined by value or var, and must only be used for UncertParameter elements, not UncertSpan elements. Definitions taken from https://web.archive.org/web/20161029215725/uncertml.org/).

- coefficientOfVariation: For a random variable with mean $\mu$ and strictly positive standard deviation $\sigma$, the coefficient of variation is defined as the ratio $\frac{\sigma}{|\mu|}$. One benefit of using the coefficient of variation rather than the standard deviation is that it is unitless.

- `kurtosis`: The kurtosis of a distribution is a measure of how peaked the distribution is. The kurtosis is defined as $\mu_4/\sigma^4$ where $\mu_4$ is the fourth central moment of the distribution and $\sigma$ is its standard deviation.
- `mean`: The arithmetic mean (typically just the mean) is what is commonly called the average. It is defined as $\bar{x} = \frac{1}{n} \cdot \sum_{i=1}^{n} x_i$ where $x_i$ represents with $i$th observation of the quantity $x$ in the sample set of size $n$. It is related to the expected value of a random variable, $\mu = E[X]$ in that the population mean, $\mu$, which is the average of all quantities in the population and is typically not known, is replaced by its estimator, the sample mean $\bar{x}$. Note that this statistic does not deal with issues of sample size, rather the mean is taken to refer to the population mean.
- `median`: The median is described as the numeric value separating the higher half of a sample (or population) from the lower half. The median of a finite list of numbers can be found by arranging all the observations from lowest value to highest value and picking the middle one. If there is an even number of observations, then there is no single middle value, then the average of the two middle values is used. The median is also the 0.5 quantile, or 50th percentile.
- `mode`: The mode is the value that occurs the most frequently in a data set (or a probability distribution). It need not be unique (e.g., two or more quantities occur equally often) and is typically defined for continuous valued quantities by first defining the histogram, and then giving the central value of the bin containing the most counts.
- `sampleSize`: The sample size is a direct count of the number of observations made or the number of samples measured. It is used in several other statistical measurements, and can be used to convert one to another.
- `skewness`: The skewness of a random variable is a measure of how asymmetric the corresponding probability distribution is. The skewness is defined as $\mu_3/\sigma^3$ where $\mu_3$ is the 3rd central moment of the distribution and $\sigma$ is its standard deviation.
- `standardDeviation`: The standard deviation of a distribution or population is the square root of its variance and is given by $\sigma = \sqrt{E[(X-\mu)^2]}$ where $\mu = E[X]$. The population standard deviation is given by $\sigma = \sqrt{\frac{1}{n} \sum_{i=1}^{n} \left(x_i - \bar{x}\right)^2}$ where $\bar{x} = \frac{1}{n} \cdot \sum_{i=1}^{n} x_i$, and $x_i$ represents the $i$th observation of the quantity $x$ in the population of size $n$. The standard deviation is a widely used measure of the variability or dispersion since it is reported in the natural units of the quantity being considered. Note that if a finite sample of a population has been used then the sample standard deviation is the appropriate unbiased estimator to use.
- `standardError`: The standard error is the standard deviation of estimates of a population value. If that population value is a mean, this statistic is called the standard error of the mean. It is calculated as the standard deviation of a sample divided by the square root of the number of the sample size. As the sample size increases, the sample size draws closer to the population size, and the standard error approaches zero. $\sigma_{\bar{x}} = \sigma/\sqrt{n}$.
- `variance`: The variance of a random quantity (or distribution) is the average value of the square of the deviation of that variable from its mean, given by $\sigma^2 = \mathrm{Var}[X] = E[(X-\mu)^2]$ where $\mu = E[X]$. The complete population variance is given by $\sigma^2 = \frac{1}{n} \sum_{i=1}^{n} \left(x_i - \bar{x}\right)^2$ where $\bar{x} = \frac{1}{n} \cdot \sum_{i=1}^{n} x_i$, and $x_i$ represents the $i$th observation of the quantity $x$ in the population of size $n$. This is the estimator of the population variance and should be replaced by the sample variance when using samples of finite size.

The following `UncertKind` values are all spans, and may only be used for **UncertSpan** elements. They are defined by an upper and lower value. Definitions taken from taken from `https://web.archive.org/web/20161029215725/uncertml.org/`).

- `confidenceInterval`: For a univariate random variable $x$, a confidence interval is a range $[a, b]$, $a < b$, so that $x$ lies between $a$ and $b$ with given probability. For example, a 95% confidence interval is a range in which

$x$ falls 95% of the time (or with probability 0.95). Confidence intervals provide intuitive summaries of the statistics of the variable $x$.

If $x$ has a continuous probability distribution $P$, then $[a, b]$ is a 95% confidence interval if $\int_a^b P(x) = 0.95$.

Unless specified otherwise, the confidence interval is usually chosen so that the remaining probability is split equally, that is $P(x < a) = P(x > b)$. If $x$ has a symmetric distribution, then the confidence intervals are usually centered around the mean. However, non-centered confidence intervals are possible and are better described by their lower and upper quantiles or levels. For example, a 50% confidence interval would usually lie between the 25% and 75% quantiles, but could in theory also lie between the 10% and 60% quantiles, although this would be rare in practice. The `confidenceInterval` allows you the flexibility to specify non-symmetric confidence intervals however in practice we would expect the main usage to be for symmetric intervals.

The `confidenceInterval` child of a **Uncertainty** is always the 95% confidence interval. For other confidence intervals, use an **UncertParameter** of type "`externalParameter`" instead.

- `credibleInterval`: In Bayesian statistics, a credible interval is similar to a confidence interval determined from the posterior distribution of a random variable $x$. That is, given a prior distribution $p(x)$ and some observations $D$, the posterior probability $p(x \mid D)$ can be computed using Bayes theorem. A 95% credible interval is then any interval $[a, b]$ so that $\int_a^b p(x \mid D) = 0.95$, that is the variable $x$ lies in the interval $[a, b]$ with posterior probability 0.95. Note that the interpretation of a credible interval is not the same as a (frequentist) confidence interval.

The `credibleInterval` child of a **Uncertainty** is always the 95% credible interval. For other credibility intervals, use an **UncertParameter** of type "`externalParameter`" instead.

- `interquartileRange`: The interquartile range is the range between the 1st and 3rd quartiles. It contains the middle 50% of the sample realisations (or of the sample probability). It is typically used and shown in box plots.

- `range`: The range is the interval $[a, b]$ so that $a < b$ and contains all possible values of $x$. This is also often called the statistical range, which is the distance from the smallest value to the largest value in a sample dataset. For a sample dataset $X = (x_1, ..., x_N)$, the range is the distance from the smallest $x_i$ to the largest. It is often used as a first estimate of the sample dispersion.

Finally, we have the "`distribution`" and "`externalParameter`" types:

- `distribution`: If the uncertainty is defined by a known distribution, that distribution may either be defined by using the child `math` element, or by using the `definitionURL`. When the `math` child is used, that math should contain the distribution in question: typically this will be a distribution `csymbol` but may be something more complicated, like a `piecewise` function. If the `definitionURL` is used, many more distributions may be used than are defined in this specification (like an `externalParameter`, below). To fully define this distributon, it will almost certainly be necessary to further define that distribution with child **UncertParameter** elements. For example, a Beta distribution takes two parameters ($\alpha$ and $\beta$), each of which could be defined by a child **UncertParameter** of type "`externalParameter`", with appropriate `definitionURL` values. A `type` of value "`distribution`" is only valid for **UncertParameter** elements, not **UncertSpan** elements.

- `externalParameter`: This type is uniquely described by an appropriate `definitionURL`, and is provided to allow a modeler to encode externally-provided parameters not otherwise explicitly handled by this specification. The range of possibilities is vast, so modelers should ensure that the tool they wish to use encodes support for any **UncertParameter** they define. As an external parameter may take any form, there are no restrictions on what other attributes or children that may be used by an **UncertParameter** of this type: it may be a single value; it may be a span; it may be defined by a child `math` element; it may be defined by child **UncertParameter** elements; it may be defined by any combination of the above. The only restriction is that the `definitionURL` *must* be defined for any **UncertParameter** of type "`externalParameter`". This type value may be used for either **UncertParameter** or **UncertSpan** elements.

As an example, here's an **UncertSpan** that defines the 99% confidence interval of a **Parameter** (using made-up `definitionURL` values):

```
<parameter id="p1" value="3.42" constant="true">
  <distrib:listOfUncertainties>
    <distrib:uncertainty>
      <distrib:uncertSpan type="externalParameter" lowerValue="3.19" upperValue="3.83"
                          definitionURL="http://dist.org/CI">
        <distrib:listOfUncertParameters>
          <distrib:uncertParameter type="externalParameter" value="0.99"
                          definitionURL="http://dist.org/CIpercent">
        </distrib:listOfUncertParameters>
      </distrib:uncertSpan>
    </distrib:uncertainty>
  </distrib:listOfUncertainties>
</parameter>
```

As examples, the following statistics are not defined by a single value nor by a range, and would therefore be good candidates for encoding as an external parameter. These terms were included in the now-defunct UncertML (and the definitions were again taken from `https://web.archive.org/web/20161029215725/uncertml.org/`), and may also be findable in other ontologies such as STATO (which has a searchable database at `https://www.ebi.ac.uk/ols/ontologies/stato`):

- `centralMoment`: For a given positive natural number $k$, the $k^{\text{th}}$ central moment of a random variable $x$ is defined as $\mu_k = E[(x - E[x])^k]$. That is, it is the expected value of the deviation from the mean to the power $k$. In particular, $\mu_0 = 1$, $\mu_1 = 0$ and $\mu_2$ is the variance of $x$.
- `correlation`: The correlation between two random variables $x_1$ and $x_2$ is the extent to which these variable vary together in a linear fashion. It is characterized by the coefficient $\rho_{1,2} = E[(x_1 - \mu_1)(x_2 - \mu_2)]/\sigma_1\sigma_2$ where $\mu_1$ and $\mu_2$ are the means of $x_1$ and $x_2$ respectively, and $\sigma_1$ and $\sigma_2$ are their respective standard deviations. Note this is strictly not a description of uncertainty, but it can be useful to represent the correlation between two variables. Generally a covariance specification would be preferred since this describes the uncertainty.
- `decile`: A decile, $d$, is any of the nine values that divide the sorted quantities into ten equal parts, so that each part represents $1/10$ of the sample, population or distribution. The first decile is equivalent to the 10th percentile.
- `moment`: For a given positive natural number $k$, the $k^{\text{th}}$ moment of a random variable $x$ is defined as $\mu_k = E[x^k]$. In particular, $\mu_0 = 1$ and $\mu_1$ is the mean of $x$. The moments can be defined with respect to some point $a$, that is $\mu_k(a) = E[(x - a)^k]$. Moments defined about the mean are called central moments.
- `percentile`: A percentile is the value of a quantity below which a certain percent of values fall. This can be defined for samples, populations and distributions. For finite samples there is no widely accepted method, but all methods essentially rank the quantities and then use some interpolation to compute the percentile, unless the sample size $n$ is a multiple of 100. For probability distributions the inverse cumulative density function can be used. The most widely used method is as follows: to estimate the value, $x_p$, of the $p$th percentile of an ascending ordered dataset containing $n$ elements with values $x_1, x_2, \ldots, x_n$ first compute $\rho = \frac{p}{100}(n-1) + 1$. Now $\rho$ is split into its integer component, $k$, and decimal component, $d$, such that $\rho = k + d$. $x_p$ is then calculated as $x_p = x_k + d(x_{k+1} - x_k)$ where $1 < \rho < n$ with special cases $x_p = x_1$ $[\rho = 1]$; $x_n$ $[\rho = n]$.
- `probability`: Given a random variable $x$ with probability density function $f(x)$, the probability that $x$ lies in some part of its domain $\mathscr{X}$ is defined as $P(x \in \mathscr{X}) = \int_{x \in \mathscr{X}} f(x)$. $\mathscr{X}$ can be defined as a lower- or upper-bounded range, e.g., $P(x < 3.2)$, or as the intersection of several such ranges, e.g., $P(x \geq 1.7 \cap x < 3.2)$.
- `quantile`: Given a random variable $x$, the $n$-quantiles are the values of $x$ which split the domain into $n$ regions of equal probability. For instance, the $k^{\text{th}}$ $n$-quantile is the value $q_k$ for which $P(x < q_k) = \frac{k}{n}$. For some common values of $n$, the $n$-quantiles have additional names, namely quartiles for $n = 4$, deciles for $n = 10$

and percentiles for $n = 100$. More generally, a quantile can be associated to any probability $p$, so that $q$ is the value of $x$ below which a proportion $p$ of the probability lies, i.e., $P(x < q) = p$. The plot on the right shows the 1st to 9th 10-quantiles (or deciles) for a normal distribution ($\mu = 4$, $\sigma = 1$) as orange dots. The blue curve is the cumulative density function of $x$. Note how the quantiles split the probability (y-axis) into 10 equal regions.

- `quartile`: The quartiles are the 4-quantiles, that is the 4 values of $x$ below which lies a proportion 0.25, 0.50, 0.75 and 1 of the probability. One can also think of them as the 4 values of $x$ which split the domain into 4 regions of equal probability.

### 3.14 The uncertainty of a Species

A **Species** is a unique SBML construct in that its value is either an amount or a concentration, depending on the value of its `hasOnlySubstanceUnits` attribute (“`true`” for amount, or “`false`” for concentration). The value of its uncertainty tracks with this: if the value of `hasOnlySubstanceUnits` on the parent **Species** is “`true`”, the uncertainty is in terms of amounts, and if “`false`”, the uncertainty is in terms of concentration.

If a **Species** is being modeled in SBML in amounts, but was measured in terms of its concentration, or visa versa, an **InitialAssignment** should be created that explicitly handles this conversion and assigns the appropriate value to the **Species**, as in the example below.

```
<listOfCompartments>
  <compartment id="C" spatialDimensions="3" size="2" constant="true">
    <distrib:listOfUncertainties>
      <distrib:uncertainty>
        <distrib:uncertParameter type="standardDeviation" value="0.15"/>
      </distrib:uncertainty>
    </distrib:listOfUncertainties>
  </compartment>
</listOfCompartments>
<listOfSpecies>
  <species id="S_amt" compartment="C" hasOnlySubstanceUnits="true"
           boundaryCondition="false" constant="false"/>
</listOfSpecies>
<listOfParameters>
  <parameter id="S_conc" value="3.4" constant="true">
    <distrib:listOfUncertainties>
      <distrib:uncertainty>
        <distrib:uncertParameter type="standardDeviation" value="0.3"/>
      </distrib:uncertainty>
    </distrib:listOfUncertainties>
  </parameter>
</listOfParameters>
<listOfInitialAssignments>
  <initialAssignment symbol="S_amt">
    <math xmlns="http://www.w3.org/1998/Math/MathML">
      <apply>
        <times/>
        <ci> S_conc </ci>
        <ci> C </ci>
      </apply>
    </math>
  </initialAssignment>
</listOfInitialAssignments>
```

Here, the uncertainty of the species “`S_amt`” is not set explicitly, and instead can be derived from the uncertainty of the values in its initial assignment (“`S_conc`” and “`C`”).

### 3.15 Examples using Uncertainty

Several examples are given to illustrate the use of the **Uncertainty** class:

#### *3.15.1 Basic Uncertainty example*

In this examples, a species is given an **Uncertainty** child to describe its standard deviation:

```
<species id="s1" compartment="C" initialAmount="3.22" hasOnlySubstanceUnits="true"
         boundaryCondition="false" constant="false">
  <distrib:listOfUncertainties>
    <distrib:uncertainty>
      <distrib:uncertParameter type="standardDeviation" distrib:value="0.3"/>
    </distrib:uncertainty>
  </distrib:listOfUncertainties>
</species>
```

Here, the species with an initial amount of 3.22 is described as having a standard deviation of 0.3, a value that might be written as "`3.22 ± 0.3`". This is probably the simplest way to use the package to introduce facts about the uncertainty of the measurements of the values present in the model.

It is also possible to include additional information about the species, should more be known:

```
<species id="s1" compartment="C" initialAmount="3.22" hasOnlySubstanceUnits="true"
         boundaryCondition="false" constant="false">
  <distrib:listOfUncertainties>
    <distrib:uncertainty>
      <distrib:listOfUncertParameters>
        <distrib:uncertParameter type="mean" distrib:value="3.2"/>
        <distrib:uncertParameter type="standardDeviation" distrib:value="0.3"/>
        <distrib:uncertParameter type="variance" distrib:value="0.09"/>
      </distrib:listOfUncertParameters>
    </distrib:uncertainty>
  </distrib:listOfUncertainties>
</species>
```

In this example, the initial amount of 3.22 is noted as having a mean of 3.2, a standard deviation of 0.3, and a variance of 0.09. Note that the standard deviation can be calculated from the variance (or visa versa), but the modeler has chosen to include both here for convenience. Note too that this use of the **Uncertainty** element does not imply that the species amount comes from a normal distribution with a mean of 3.2 and standard deviation of 0.3, but rather that the species amount comes from an unknown distribution with those qualities. If it is known that the value was drawn from a particular distribution, an **UncertParameter** of type "`distribution`" should be used, rather than **UncertParameter** elements of type "`mean`" and "`standardDeviation`".

Note also that 3.22 (the `initialAmount`) is different from 3.2 (the `mean`): evidently, this model was constructed as a realization of the underlying uncertainty, instead of simply using the mean.

#### *3.15.2 Defining a random variable*

In addition to describing the uncertainty about an experimental observation one can also use this mechanism to describe a parameter as a random variable. In the example below the parameter, `Z`, is defined as following a gamma distribution, with a given shape and scale. No value is given for the parameter so it is then up the modeler to decide how to use this random variable. For example they may choose to simulate the model in which case they may provide values for `shape_Z` and `scale_Z` and then sample a random value from the simulation. Alternatively they may choose to carry out a parameter estimation and use experimental observations to estimate `shape_Z` and `scale_Z`.

For added information, the modeler has chosen to include the observed mean and variance of the value. These are close to the expected mean and variance from the given distribution (1.0 and 0.1, respectively, given the shape and scale), but were slightly different due to the sample size.

```
<listOfParameters>
  <parameter id="shape_Z" value="10" constant="true"/>
  <parameter id="scale_Z" value="0.1" constant="true"/>
  <parameter id="Z" constant="true">
    <distrib:listOfUncertainties>
      <distrib:uncertainty>
        <distrib:uncertParameter type="distribution">
          <math xmlns="http://www.w3.org/1998/Math/MathML">
            <apply>
              <csymbol definitionURL="http://www.sbml.org/sbml/symbols/distrib/gamma"
                       encoding="text"> gamma </csymbol>
              <ci> shape_Z </ci>
              <ci> scale_Z </ci>
            </apply>
          </math>
        </distrib:uncertParameter>
        <distrib:uncertParameter type="mean" value="1.03"/>
        <distrib:uncertParameter type="variance" value="0.97"/>
      </distrib:uncertainty>
    </distrib:listOfUncertainties>
  </parameter>
</listOfParameters>
```

#### 3.15.3 Defining external distributions

If an SBML value is drawn from a distribution not defined explicitly in this specification, it is necessary to use an **UncertParameter** of type “`externalParameter`” to define the distribution’s parameters. In this example, the parameter `p1` was drawn from a zeta distribution, with a shape parameter of 2.37. An **UncertParameter** of type “`distribution`” is created with the ’zeta’ URI, with a child **UncertParameter** of type “`externalParameter`” with the ’shape’ URI for its `definitionURL`. For readability, ’zeta’ and ’shape’ were used as the names of these parameters.

```
<parameter id="p1" constant="true">
  <listOfUncertainties xmlns="http://www.sbml.org/sbml/level3/version1/distrib/version1">
    <uncertainty>
      <uncertParameter type="distribution" name="zeta"
              definitionURL="http://www.probonto.org/ontology#PROB_k0001263">
        <listOfUncertParameters>
          <uncertParameter type="externalParameter" name="shape" value="2.37"
              definitionURL="http://purl.obolibrary.org/obo/STATO_0000436"/>
        </listOfUncertParameters>
      </uncertParameter>
    </uncertainty>
  </listOfUncertainties>
</parameter>
```

It is also possible to create even more complex structures with the **UncertParameter** scheme. In this example, we define a categorical distribution based on data from three patients. The parent **UncertParameter** is defined to be the ’categorical’ distribution, with three ’category’ children, each with two child ’value’ and ’probability’ parameters. Collectively, they define a distribution where a value of 1.01 has a probability of 50%, a value of 2.24 has a probability of 25%, and a value of 1.72 has a probability of 25%. (The `definitionURL` examples here were made up for the purposes of this example, to be readable. In an actual SBML document, they would point to existing external ontologies.)

```
<listOfUncertainties xmlns="http://www.sbml.org/sbml/level3/version1/distrib/version1">
  <uncertainty>
    <listOfUncertParameters>
      <uncertParameter type="distribution" definitionURL="http://dist.org/categorical">
        <listOfUncertParameters>
          <uncertParameter type="externalParameter" id="p1" definitionURL="http://dist.org/category">
            <listOfUncertParameters>
              <uncertParameter type="externalParameter" value="1.01"
                               definitionURL="http://dist.org/cat_val"/>
              <uncertParameter type="externalParameter" value="0.5"
                               definitionURL="http://dist.org/cat_prob"/>
            </listOfUncertParameters>
          </uncertParameter>
          <uncertParameter type="externalParameter" id="p2" definitionURL="http://dist.org/category">
            <listOfUncertParameters>
              <uncertParameter type="externalParameter" value="2.24"
                               definitionURL="http://dist.org/cat_val"/>
              <uncertParameter type="externalParameter" value="0.25"
                               definitionURL="http://dist.org/cat_prob"/>
            </listOfUncertParameters>
          </uncertParameter>
          <uncertParameter type="externalParameter" id="p3" definitionURL="http://dist.org/category">
            <listOfUncertParameters>
              <uncertParameter type="externalParameter" value="1.72"
                               definitionURL="http://dist.org/cat_val"/>
              <uncertParameter type="externalParameter" value="0.25"
                               definitionURL="http://dist.org/cat_prob"/>
            </listOfUncertParameters>
          </uncertParameter>
        </listOfUncertParameters>
      </uncertParameter>
    </listOfUncertParameters>
  </uncertainty>
</listOfUncertainties>
```

## 4 Interaction with other packages

### 4.1 Custom annotations for function definitions

Before this package was available, a collection of SBML simulator authors developed an *ad hoc* convention for exchanging annotated **FunctionDefinition** objects that represented draws from distributions. This convention is described at http://co.mbine.org/specifications/sbml.proposal.distrib-annotations.version-1 by Frank T. Bergmann, and represents a basic starting point for any modeler interested in exchanging SBML models containing draws from distributions.

When implementing Distributions support, it would be possible to include “backwards” support for this annotation convention by wrapping any calls to a distribution in a **FunctionDefinition**, and annotating that using this scheme.

Table 4 is taken from the above document by Frank Bergmann, and can be used as a template if translating from that **FunctionDefinition** system to the Distributions extended **Math** system. The suggested fallback function returns the mean of the distribution.

***Table 4:*** *The annotation URLs.*

| Id | Distribution | Definition URL | Fallback |
|---|---|---|---|
| uniform | Uniform | http://en.wikipedia.org/wiki/Uniform_distribution_(continuous) | $lambda(a, b, \frac{a+b}{2})$ |
| normal | Normal | http://en.wikipedia.org/wiki/Normal_distribution | $lambda(m, s, m)$ |
| exponential | Exponential | http://en.wikipedia.org/wiki/Exponential_distribution | $lambda(l, 1/l)$ |
| gamma | Gamma | http://en.wikipedia.org/wiki/Gamma_distribution | $lambda(a, b, a \times b)$ |
| poisson | Poisson | http://en.wikipedia.org/wiki/Poisson_distribution | $lambda(\mu, \mu)$ |
| lognormal | Lognormal | http://en.wikipedia.org/wiki/Log-normal_distribution | $lambda(z, s, e^{z+s^2/2})$ |
| chisq | Chi-squared | http://en.wikipedia.org/wiki/Chi-squared_distribution | $lambda(\nu, \nu)$ |
| laplace | Laplace | http://en.wikipedia.org/wiki/Laplace_distribution | $lambda(a, 0)$ |
| cauchy | Cauchy | http://en.wikipedia.org/wiki/Cauchy_distribution | $lambda(a, a)$ |
| rayleigh | Rayleigh | http://en.wikipedia.org/wiki/Rayleigh_distribution | $lambda(s, s \times \sqrt{\pi/2})$ |
| binomial | Binomial | http://en.wikipedia.org/wiki/Binomial_distribution | $lambda(p, n, p \times n)$ |
| bernoulli | Bernoulli | http://en.wikipedia.org/wiki/Bernoulli_distribution | $lambda(p, p)$ |

As an example, here is a complete (if small) model that uses the above “custom annotation” scheme:

```
<?xml version="1.0" encoding="UTF-8"?>
<sbml xmlns="http://www.sbml.org/sbml/level3/version1/core"
      level="3" version="1">
  <model>
    <listOfFunctionDefinitions>
      <functionDefinition id="normal">
        <annotation>
          <distribution xmlns="http://sbml.org/annotations/distribution"
                   definition="http://en.wikipedia.org/wiki/Normal_distribution"/>
        </annotation>
        <math xmlns="http://www.w3.org/1998/Math/MathML">
          <lambda>
            <bvar>
              <ci> mean </ci>
            </bvar>
            <bvar>
```

```
            <ci> stdev </ci>
          </bvar>
          <notanumber/>
        </lambda>
      </math>
    </functionDefinition>
  </listOfFunctionDefinitions>
  <listOfParameters>
    <parameter id="x" constant="true"/>
  </listOfParameters>
  <listOfInitialAssignments>
    <initialAssignment symbol="x">
      <math xmlns="http://www.w3.org/1998/Math/MathML">
        <apply>
          <ci> normal </ci>
          <cn> 3 </cn>
          <cn> 0.2 </cn>
        </apply>
      </math>
    </initialAssignment>
  </listOfInitialAssignments>
 </model>
</sbml>
```

And here is the same model, using the `csymbol` defined in *distrib*:

```
<?xml version="1.0" encoding="UTF-8"?>
<sbml xmlns="http://www.sbml.org/sbml/level3/version2/core"
      xmlns:distrib="http://www.sbml.org/sbml/level3/version1/distrib/version1"
      level="3" version="2" distrib:required="true">
  <model>
    <listOfParameters>
      <parameter id="x" constant="true"/>
    </listOfParameters>
    <listOfInitialAssignments>
      <initialAssignment symbol="x">
        <math xmlns="http://www.w3.org/1998/Math/MathML">
          <apply>
              <csymbol definitionURL="http://www.sbml.org/sbml/symbols/distrib/normal"
                       encoding="text"> normal </csymbol>
            <cn type="integer"> 3 </cn>
            <cn> 0.2 </cn>
          </apply>
        </math>
      </initialAssignment>
    </listOfInitialAssignments>
  </model>
</sbml>
```

### 4.2 The Arrays package

This package is dependent on no other package, but might rely on the Arrays package to provide vector and matrix structures if those are desired/used. Note that currently, the only case where arrays could be used is when an **UncertParameter** of type "`externalParameter`" is defined that requires array input or output.

### 4.3 SBML Level 3 Version 2

This package may be used with either SBML Level 3 Version 1 Core, or SBML Level 3 Version 2 Core, and no construct in this package changes as a result: the addition of `id` and `name` to **DistribBase** means that the addition of those attributes to ***SBase*** in SBML Level 3 Version 2 Core is redundant.

Another change between SBML Level 3 Version 1 and Version 2 is that in Version 2, core elements and core **Math**

may refer to package `ids` with mathematical meaning. However, Distributions **UncertParameter** elements do not have mathematical meaning, and may not be used in this fashion. Instead, the `var` attribute should be used to connect the element to a core **Parameter**, instead of using the `value` attribute. This approach has the advantage of working both in Version 1 and Version 2 of SBML Core.

### 4.4 Other SBML Level 3 Packages

This package may be used seamlessly with other SBML Level 3 packages that have **Math** elements, and/or that have elements with mathematical meaning. It would be possible, for example, to use a *distrib* `csymbol` in the **Math** of a 'Qualitative Models' `<functionTerm>`. In this example, the `functionTerm` returns true when a quantity 'A' is greater than or equal to a value drawn from a uniform distribution:

```
<qual:transition qual:id="tr_B">
  [...]
  <qual:listOfFunctionTerms>
    <qual:functionTerm qual:resultLevel="1">
      <math xmlns="http://www.w3.org/1998/Math/MathML">
        <!-- A >= uniform(0,2)-->
        <apply>
          <geq/>
          <ci>A</ci>
          <apply>
            <csymbol definitionURL="http://www.sbml.org/sbml/symbols/distrib/uniform"
                     encoding="text"> uniform </csymbol>
            <cn type="integer"> 0 </cn>
            <cn type="integer"> 2 </cn>
          </apply>
        </apply>
      </math>
    </qual:functionTerm>
    <qual:defaultTerm qual:resultLevel="0"/>
  </qual:listOfFunctionTerms>
</qual:transition>
```

A Qualitative Species could also be given a child **Uncertainty**. Here, the value of A is described as coming from a poisson distribution:

```
<qual:qualitativeSpecies qual:compartment="cytosol" qual:constant="false"
                         qual:id="A" qual:maxLevel="6">
  <distrib:listOfUncertainties>
    <distrib:uncertainty>
      <distrib:uncertParameter distrib:type="distribution">
        <math xmlns="http://www.w3.org/1998/Math/MathML">
          <apply>
            <csymbol definitionURL="http://www.sbml.org/sbml/symbols/distrib/poisson"
                     encoding="text"> poisson </csymbol>
            <cn type="integer"> 0 </cn>
          </apply>
        </math>
      </distrib:uncertParameter>
    </distrib:uncertainty>
  </distrib:listOfUncertainties>
</qual:qualitativeSpecies>
```

These constructs can be used in identical ways in other SBML Level 3 packages.

## 5 Use-cases and examples

The following examples are more fleshed out than the ones in the main text, and/or illustrate features of this package that were not previously illustrated.

### 5.1 Sampling from a distribution: PK/PD Model

This is a very straightforward use of a log normal distribution. The key point to note is that a value is sampled from the distribution and assigned to a variable when it is invoked in the `initialAssignment` elements in this example. Later use of the variable does not result in re-sampling from the distribution. This is consistent with current SBML semantics.

```
<?xml version="1.0" encoding="UTF-8"?>
<sbml xmlns="http://www.sbml.org/sbml/level3/version1/core"
  xmlns:distrib="http://www.sbml.org/sbml/level3/version1/distrib/version1"
  level="3" version="1" distrib:required="true">
  <model>
    <listOfCompartments>
      <compartment id="central" size="0" constant="true"/>
      <compartment id="gut" size="0" constant="true"/>
    </listOfCompartments>
    <listOfSpecies>
      <species id="Qc" compartment="central" initialAmount="1" hasOnlySubstanceUnits="true"
        boundaryCondition="false" constant="false"/>
      <species id="Qg" compartment="gut" initialAmount="1" hasOnlySubstanceUnits="true"
        boundaryCondition="false" constant="false"/>
    </listOfSpecies>
    <listOfParameters>
      <parameter id="ka" constant="true"/>
      <parameter id="ke" constant="true"/>
      <parameter id="Cc" constant="false"/>
      <parameter id="Cc_obs" constant="false"/>
    </listOfParameters>
    <listOfInitialAssignments>
      <initialAssignment symbol="central">
        <math xmlns="http://www.w3.org/1998/Math/MathML">
          <apply>
            <csymbol encoding="text"
              definitionURL="http://www.sbml.org/sbml/symbols/distrib/lognormal"> lognormal
            </csymbol>
            <cn> 0.5 </cn>
            <cn> 0.1 </cn>
          </apply>
        </math>
      </initialAssignment>
      <initialAssignment symbol="ka">
        <math xmlns="http://www.w3.org/1998/Math/MathML">
          <apply>
            <csymbol encoding="text"
              definitionURL="http://www.sbml.org/sbml/symbols/distrib/lognormal"> lognormal
            </csymbol>
            <cn> 0.5 </cn>
            <cn> 0.1 </cn>
          </apply>
        </math>
      </initialAssignment>
      <initialAssignment symbol="ke">
        <math xmlns="http://www.w3.org/1998/Math/MathML">
          <apply>
            <csymbol encoding="text"
              definitionURL="http://www.sbml.org/sbml/symbols/distrib/lognormal"> lognormal
            </csymbol>
```

```
            <cn> 0.5 </cn>
            <cn> 0.1 </cn>
          </apply>
        </math>
      </initialAssignment>
    </listOfInitialAssignments>
    <listOfRules>
      <assignmentRule variable="Cc">
        <math xmlns="http://www.w3.org/1998/Math/MathML">
          <apply>
            <divide/>
            <ci> Qc </ci>
            <ci> central </ci>
          </apply>
        </math>
      </assignmentRule>
      <assignmentRule variable="Cc_obs">
        <math xmlns="http://www.w3.org/1998/Math/MathML">
          <apply>
            <plus/>
            <ci> Cc </ci>
            <cn type="integer"> 1 </cn>
          </apply>
        </math>
      </assignmentRule>
    </listOfRules>
    <listOfReactions>
      <reaction id="absorption" reversible="false" fast="false">
        <listOfReactants>
          <speciesReference species="Qg" stoichiometry="1" constant="true"/>
        </listOfReactants>
        <listOfProducts>
          <speciesReference species="Qc" stoichiometry="1" constant="true"/>
        </listOfProducts>
        <kineticLaw>
          <math xmlns="http://www.w3.org/1998/Math/MathML">
            <apply>
              <times/>
              <ci> ka </ci>
              <ci> Qg </ci>
            </apply>
          </math>
        </kineticLaw>
      </reaction>
      <reaction id="excretion" reversible="false" fast="false">
        <listOfReactants>
          <speciesReference species="Qc" stoichiometry="1" constant="true"/>
        </listOfReactants>
        <kineticLaw>
          <math xmlns="http://www.w3.org/1998/Math/MathML">
            <apply>
              <divide/>
              <apply>
                <times/>
                <ci> ke </ci>
                <ci> Qc </ci>
              </apply>
              <ci> central </ci>
            </apply>
          </math>
        </kineticLaw>
      </reaction>
    </listOfReactions>
  </model>
</sbml>
```

### 5.2 Multiple uses of distributions

In this example, a `normal` csymbol is used in an initial assignment, and `mean` and `standardDeviation` elements are used to denote the uncertainty in the parameter `V`, and the uncertainty in the initial assignment to `V`. Note that strictly speaking, one could assume that the uncertainty in the parameter itself was identical to the uncertainty in its initial assignment; both are given here by way of illustration.

```
<?xml version="1.0" encoding="UTF-8"?>
<sbml xmlns="http://www.sbml.org/sbml/level3/version1/core"
  xmlns:distrib="http://www.sbml.org/sbml/level3/version1/distrib/version1"
  level="3" version="1" distrib:required="true">
  <model>
    <listOfParameters>
      <parameter id="V" constant="true">
        <distrib:listOfUncertainties>
          <distrib:uncertainty>
            <distrib:uncertParameter distrib:var="V_pop" distrib:type="mean"/>
            <distrib:uncertParameter distrib:var="V_omega" distrib:type="standardDeviation"/>
          </distrib:uncertainty>
        </distrib:listOfUncertainties>
      </parameter>
      <parameter id="V_pop" value="100" constant="true"/>
      <parameter id="V_omega" value="0.25" constant="true"/>
    </listOfParameters>
    <listOfInitialAssignments>
      <initialAssignment symbol="V">
        <math xmlns="http://www.w3.org/1998/Math/MathML">
          <apply>
            <csymbol encoding="text"
              definitionURL="http://www.sbml.org/sbml/symbols/distrib/normal"> normal
            </csymbol>
            <ci> V_pop </ci>
            <ci> V_omega </ci>
          </apply>
        </math>
        <distrib:listOfUncertainties>
          <distrib:uncertainty>
            <distrib:uncertParameter distrib:var="V_pop" distrib:type="mean"/>
            <distrib:uncertParameter distrib:var="V_omega" distrib:type="standardDeviation"/>
          </distrib:uncertainty>
        </distrib:listOfUncertainties>
      </initialAssignment>
    </listOfInitialAssignments>
  </model>
</sbml>
```

### 5.3 Defining confidence intervals

In this example, several **Parameter** elements are given confidence intervals, and several **Species** are given standard deviations. Each indicates the modeler's assessment of the precision of the estimated given values for those elements.

```
<?xml version="1.0" encoding="UTF-8"?>
<sbml xmlns="http://www.sbml.org/sbml/level3/version1/core" level="3" version="1"
      xmlns:distrib="http://www.sbml.org/sbml/level3/version1/distrib/version1"
      distrib:required="true">
  <model>
    <listOfCompartments>
      <compartment id="C" spatialDimensions="3" size="1" constant="true"/>
    </listOfCompartments>
    <listOfSpecies>
      <species id="S1" compartment="C" initialAmount="5.2" hasOnlySubstanceUnits="false"
```

```xml
        boundaryCondition="false" constant="false">
        <distrib:listOfUncertainties>
          <distrib:uncertainty>
            <distrib:uncertParameter distrib:value="0.3" distrib:type="standardDeviation"/>
          </distrib:uncertainty>
        </distrib:listOfUncertainties>
      </species>
      <species id="S2" compartment="C" initialAmount="8.7" hasOnlySubstanceUnits="false"
        boundaryCondition="false" constant="false">
        <distrib:listOfUncertainties>
          <distrib:uncertainty>
            <distrib:uncertParameter distrib:value="0.01" distrib:type="standardDeviation"/>
          </distrib:uncertainty>
        </distrib:listOfUncertainties>
      </species>
      <species id="S3" compartment="C" initialAmount="1102" hasOnlySubstanceUnits="false"
        boundaryCondition="false" constant="false">
        <distrib:listOfUncertainties>
          <distrib:uncertainty>
            <distrib:uncertParameter distrib:value="53" distrib:type="standardDeviation"/>
          </distrib:uncertainty>
        </distrib:listOfUncertainties>
      </species>
      <species id="S4" compartment="C" initialAmount="0.026" hasOnlySubstanceUnits="false"
        boundaryCondition="false" constant="false">
        <distrib:listOfUncertainties>
          <distrib:uncertainty>
            <distrib:uncertParameter distrib:value="0.004" distrib:type="standardDeviation"/>
          </distrib:uncertainty>
        </distrib:listOfUncertainties>
      </species>
    </listOfSpecies>
    <listOfParameters>
      <parameter id="P1" value="5.13" constant="true">
        <distrib:listOfUncertainties>
          <distrib:uncertainty>
            <distrib:uncertSpan distrib:type="confidenceInterval"
                distrib:valueLower="5" distrib:valueUpper="5.32"/>
          </distrib:uncertainty>
        </distrib:listOfUncertainties>
      </parameter>
      <parameter id="P2" value="15" constant="true">
        <distrib:listOfUncertainties>
          <distrib:uncertainty>
            <distrib:uncertSpan distrib:type="confidenceInterval"
                distrib:valueLower="10.22" distrib:valueUpper="15.02"/>
          </distrib:uncertainty>
        </distrib:listOfUncertainties>
      </parameter>
      <parameter id="P3" value="0.003" constant="true">
        <distrib:listOfUncertainties>
          <distrib:uncertainty>
            <distrib:uncertSpan distrib:type="confidenceInterval"
                distrib:valueLower="-0.001" distrib:valueUpper="0.0041"/>
          </distrib:uncertainty>
        </distrib:listOfUncertainties>
      </parameter>
      <parameter id="P4" value="0.34" constant="true">
        <distrib:listOfUncertainties>
          <distrib:uncertainty>
            <distrib:uncertSpan distrib:type="confidenceInterval"
                distrib:valueLower="0.22" distrib:valueUpper="0.51"/>
          </distrib:uncertainty>
        </distrib:listOfUncertainties>
      </parameter>
      <parameter id="P5" value="92" constant="true">
```

```
        <distrib:listOfUncertainties>
          <distrib:uncertainty>
            <distrib:uncertSpan distrib:type="confidenceInterval"
                distrib:valueLower="90" distrib:valueUpper="99"/>
          </distrib:uncertainty>
        </distrib:listOfUncertainties>
      </parameter>
    </listOfParameters>
  </model>
</sbml>
```

## A Validation of SBML documents

### A.1 Validation and consistency rules

This section summarizes all the conditions that must (or in some cases, at least *should*) be true of an SBML Level 3 Version 1 model that uses the Distributions package. We use the same conventions as are used in the SBML Level 3 Version 1 Core specification document. In particular, there are different degrees of rule strictness. Formally, the differences are expressed in the statement of a rule: either a rule states that a condition *must* be true, or a rule states that it *should* be true. Rules of the former kind are strict SBML validation rules—a model encoded in SBML must conform to all of them in order to be considered valid. Rules of the latter kind are consistency rules. To help highlight these differences, we use the following three symbols next to the rule numbers:

☑ A checked box indicates a *requirement* for SBML conformance. If a model does not follow this rule, it does not conform to the Distributions package specification. (Mnemonic intention behind the choice of symbol: "This must be checked.")

▲ A triangle indicates a *recommendation* for model consistency. If a model does not follow this rule, it is not considered strictly invalid as far as the Distributions package specification is concerned; however, it indicates that the model contains a physical or conceptual inconsistency. (Mnemonic intention behind the choice of symbol: "This is a cause for warning.")

★ A star indicates a strong recommendation for good modeling practice. This rule is not strictly a matter of SBML encoding, but the recommendation comes from logical reasoning. As in the previous case, if a model does not follow this rule, it is not strictly considered an invalid SBML encoding. (Mnemonic intention behind the choice of symbol: "You're a star if you heed this.")

The validation rules listed in the following subsections are all stated or implied in the rest of this specification document. They are enumerated here for convenience. Unless explicitly stated, all validation rules concern objects and attributes specifically defined in the Distributions package package.

☞ For convenience and brevity, we use the shorthand "`distrib:x`" to stand for an attribute or element name `x` in the namespace for the Distributions package package, using the namespace prefix `distrib`. In reality, the prefix string may be different from the literal "`distrib`" used here (and indeed, it can be any valid XML namespace prefix that the modeler or software chooses). We use "`distrib:x`" because it is shorter than to write a full explanation everywhere we refer to an attribute or element in the Distributions package namespace.

Attributes from this package are listed in these rules as having the "`distrib:`" prefix, but as is convention for SBML packages, this prefix is optional.

#### *General rules about this package*

**distrib-10101** ☑ To conform to the Distributions package specification for SBML Level 3 Version 1, an SBML document must declare "`http://www.sbml.org/sbml/level3/version1/distrib/version1`" as the XMLNamespace to use for elements of this package. (Reference: SBML Level 3 Specification for Distributions, Version 1 Section 3.2 on page 10.)

**distrib-10102** ☑ Wherever they appear in an SBML document, elements and attributes from the Distributions package must use "`http://www.sbml.org/sbml/level3/version1/distrib/version1`" as the namespace, declaring so either explicitly or implicitly. (Reference: SBML Level 3 Specification for Distributions, Version 1 Section 3.2 on page 10.)

#### *General rules for MathML content*

**distrib-10205** ☑ (Extends validation rule #10205 in the SBML Level 3 Core specification.) The allowed values for the attribute `definitionURL` on a `csymbol` are extended to additionally allow

"http://www.sbml.org/sbml/symbols/distrib/normal",
"http://www.sbml.org/sbml/symbols/distrib/uniform",
"http://www.sbml.org/sbml/symbols/distrib/bernoulli",
"http://www.sbml.org/sbml/symbols/distrib/binomial",
"http://www.sbml.org/sbml/symbols/distrib/cauchy",
"http://www.sbml.org/sbml/symbols/distrib/chisquare",
"http://www.sbml.org/sbml/symbols/distrib/exponential",
"http://www.sbml.org/sbml/symbols/distrib/gamma",
"http://www.sbml.org/sbml/symbols/distrib/laplace",
"http://www.sbml.org/sbml/symbols/distrib/lognormal",
"http://www.sbml.org/sbml/symbols/distrib/poisson", and
"http://www.sbml.org/sbml/symbols/distrib/rayleigh". (Reference: SBML Level 3 Specification for Distributions, Version 1, Section 3.5 on page 12)

**distrib-10250** ☑ Any MathML `csymbol` element with a `definitionURL` of "http://www.sbml.org/sbml/symbols/distrib/normal", "http://www.sbml.org/sbml/symbols/distrib/binomial", "http://www.sbml.org/sbml/symbols/distrib/gamma", or "http://www.sbml.org/sbml/symbols/distrib/lognormal" must have exactly two or four children. (Reference: SBML Level 3 Specification for Distributions, Version 1, Section 3.5 on page 12)

**distrib-10251** ☑ Any MathML `csymbol` element with a `definitionURL` of "http://www.sbml.org/sbml/symbols/distrib/uniform" must have exactly two children. (Reference: SBML Level 3 Specification for Distributions, Version 1, Section 3.5 on page 12)

**distrib-10252** ☑ Any MathML `csymbol` element with a `definitionURL` of "http://www.sbml.org/sbml/symbols/distrib/bernoulli", must have exactly one child. (Reference: SBML Level 3 Specification for Distributions, Version 1, Section 3.5 on page 12)

**distrib-10253** ☑ Any MathML `csymbol` element with a `definitionURL` of "http://www.sbml.org/sbml/symbols/distrib/cauchy" or "http://www.sbml.org/sbml/symbols/distrib/laplace" must have exactly one, two, or four children. (Reference: SBML Level 3 Specification for Distributions, Version 1, Section 3.5 on page 12)

**distrib-10254** ☑ Any MathML `csymbol` element with a `definitionURL` of "http://www.sbml.org/sbml/symbols/distrib/chisquare", "http://www.sbml.org/sbml/symbols/distrib/exponential", "http://www.sbml.org/sbml/symbols/distrib/poisson", or "http://www.sbml.org/sbml/symbols/distrib/rayleigh" must have exactly one or three children. (Reference: SBML Level 3 Specification for Distributions, Version 1, Section 3.5 on page 12)

#### *General rules about identifiers*

**distrib-10301** ☑ (Extends validation rule #10301 in the SBML Level 3 Core specification.) The `distrib:id` attribute of every **ListOfUncertainties**, **Uncertainty**, **UncertParameter**, and **UncertSpan** is added to the general SId namespace of the **Model**, and must be unique among all core or other package `id` values also added to the same namespace. (Reference: SBML Level 3 Version 1 Core, Section 3.1.7.)

**distrib-10302** ☑ The value of a `distrib:id` must conform to the syntax of the **SBML** data type `SId` (Reference: SBML Level 3 Specification for Distributions, Version 1, Section 3.8 on page 17.)

**distrib-10303** ☑ The value of a `distrib:name` must have a value of data type `string`. (Reference: SBML Level 3 Specification for Distributions, Version 1, Section 3.8 on page 17.)

#### *Rules for the extended SBML class*

**distrib-20101** ☑ In all SBML documents using the Distributions package, the **SBML** object must have the `distrib:required` attribute. (Reference: SBML Level 3 Version 1 Core, Section 4.1.2.)

**distrib-20102** ☑ The value of attribute `distrib:required` on the **SBML** object must be of data type `boolean`. (Reference: SBML Level 3 Version 1 Core, Section 4.1.2.)

**distrib-20103** ☑ The value of attribute `distrib:required` on the **SBML** object must be set to "`true`". (Reference: SBML Level 3 Specification for Distributions, Version 1 Section 3.2 on page 10.)

#### *Rules for extended SBase object*

**distrib-20201** ☑ An ***SBase*** object may contain one and only one instance of the **ListOfUncertainties** element. No other elements from the SBML Level 3 Distributions namespaces are permitted on an ***SBase*** object. (Reference: SBML Level 3 Specification for Distributions, Version 1, Section 3.9 on page 17.)

**distrib-20202** ☑ Apart from the general notes and annotations subobjects permitted on all SBML objects, a **ListOfUncertainties** container object may only contain **Uncertainty** objects. (Reference: SBML Level 3 Specification for Distributions, Version 1, Section 3.9 on page 17.)

**distrib-20203** ☑ A **ListOfUncertainties** object may have the optional SBML Level 3 Core attributes `metaid` and `sboTerm`. No other attributes from the SBML Level 3 Core namespaces are permitted on a **ListOfUncertainties** object. (Reference: SBML Level 3 Specification for Distributions, Version 1, Section 3.9 on page 17.)

#### *Rules for UncertParameter object*

**distrib-20301** ☑ An **UncertParameter** object may have the optional SBML Level 3 Core attributes `metaid` and `sboTerm`. No other attributes from the SBML Level 3 Core namespaces are permitted on an **UncertParameter**. (Reference: SBML Level 3 Version 1 Core, Section 3.2.)

**distrib-20302** ☑ An **UncertParameter** object may have the optional SBML Level 3 Core subobjects for notes and annotations. No other elements from the SBML Level 3 Core namespaces are permitted on an **UncertParameter**. (Reference: SBML Level 3 Version 1 Core, Section 3.2.)

**distrib-20303** ☑ An **UncertParameter** object must have the required attribute `distrib:type`, and may have the optional attributes `distrib:id`, `distrib:name`, `distrib:value`, `distrib:var`, `distrib:units` and `distrib:definitionURL`. No other attributes from the SBML Level 3 Distributions namespaces are permitted on an **UncertParameter** object. (Reference: SBML Level 3 Specification for Distributions, Version 1, Section 3.11 on page 19.)

**distrib-20304** ☑ An **UncertParameter** object may contain one and only one instance of each of the **ListOfUncertParameters** and ASTNode elements. No other elements from the SBML Level 3 Distributions namespaces are permitted on an **UncertParameter** object. (Reference: SBML Level 3 Specification for Distributions, Version 1, Section 3.11 on page 19.)

**distrib-20305** ☑ The value of the attribute `distrib:type` of an **UncertParameter** object may only be a subset of the values allowed in an SBML data type `UncertType`; that is, the value must be one of the following: "`distribution`", "`externalParameter`", "`coeffientOfVariation`", "`kurtosis`", "`mean`", "`median`", "`mode`", "`sampleSize`", "`skewness`", "`standardDeviation`", "`standardError`", or "`variance`". (Reference: SBML Level 3 Specification for Distributions, Version 1, Section 3.11 on page 19.)

**distrib-20306** ☑ The attribute `distrib:value` on an **UncertParameter** must have a value of data type `double`. (Reference: SBML Level 3 Specification for Distributions, Version 1, Section 3.11 on page 19.)

**distrib-20307** ☑ The value of the attribute `distrib:var` of an **UncertParameter** object must be the identifier of an existing object derived from the ***SBase*** class and defined in the enclosing **Model** object. (Reference: SBML Level 3 Specification for Distributions, Version 1, Section 3.11 on page 19.)

**distrib-20308** ☑ The value of the attribute `distrib:units` on an **UncertParameter** must be taken from the following: the identifier of a **UnitDefinition** object in the enclosing **Model**, or one of the base units in SBML. (Reference: SBML Level 3 Specification for Distributions, Version 1, Section 3.11 on page 19.)

**distrib-20309** ☑ The attribute `distrib:definitionURL` on an **UncertParameter** must have a value of data type `string`. (Reference: SBML Level 3 Specification for Distributions, Version 1, Section 3.11 on page 19.)

**distrib-20310** ☑ Apart from the general notes and annotations subobjects permitted on all SBML objects, a **ListOfUncertParameters** container object may only contain **UncertParameter** objects. (Reference: SBML Level 3 Specification for Distributions, Version 1, Section 3.11 on page 19.)

**distrib-20311** ☑ A **ListOfUncertParameters** object may have the optional SBML Level 3 Core attributes `metaid` and `sboTerm`. No other attributes from the SBML Level 3 Core namespaces are permitted on a **ListOfUncertParameters** object. (Reference: SBML Level 3 Specification for Distributions, Version 1, Section 3.11 on page 19.)

**distrib-20312** ☑ A **ListOfUncertParameters** object may have the optional attributes `distrib:id` and `distrib:name`. No other attributes from the SBML Level 3 Distributions namespaces are permitted on an **UncertSpan** object. (Reference: SBML Level 3 Specification for Distributions, Version 1, Section 3.11 on page 19.)

**distrib-20350** ☑ An **UncertParameter** object may define either the attribute `distrib:value` or `distrib:var`, but not both. (Reference: SBML Level 3 Specification for Distributions, Version 1, Section 3.11 on page 19.)

**distrib-20351** ☑ An **UncertParameter** object may define either the attribute `distrib:value` or `distrib:var`, but not both. (Reference: SBML Level 3 Specification for Distributions, Version 1, Section 3.11 on page 19.)

**distrib-20352** ☑ An **UncertParameter** object with a `type` of "`coeffientOfVariation`", "`kurtosis`", "`mean`", "`median`", "`mode`", "`sampleSize`", "`skewness`", "`standardDeviation`", "`standardError`", or "`variance`" may not define the attribute `distrib:definitionURL`. (Reference: SBML Level 3 Specification for Distributions, Version 1, Section 3.11 on page 19.)

**distrib-20353** ☑ An **UncertParameter** object with a `type` of "`coeffientOfVariation`", "`kurtosis`", "`mean`", "`median`", "`mode`", "`sampleSize`", "`skewness`", "`standardDeviation`", "`standardError`", or "`variance`" may not define a child **Math** or **ListOfUncertParameters** object. (Reference: SBML Level 3 Specification for Distributions, Version 1, Section 3.11 on page 19.)

**distrib-20354** ☑ An **UncertParameter** object with a `type` of "`distribution`" may not define the attributes `distrib:value` or `distrib:var`. (Reference: SBML Level 3 Specification for Distributions, Version 1, Section 3.11 on page 19.)

#### *Rules for Uncertainty object*

**distrib-20401** ☑ An **Uncertainty** object may have the optional SBML Level 3 Core attributes `metaid` and `sboTerm`. No other attributes from the SBML Level 3 Core namespaces are permitted on an **Uncertainty**. (Reference: SBML Level 3 Version 1 Core, Section 3.2.)

**distrib-20402** ☑ An **Uncertainty** object may have the optional SBML Level 3 Core subobjects for notes and annotations. No other elements from the SBML Level 3 Core namespaces are permitted on an **Uncertainty**. (Reference: SBML Level 3 Version 1 Core, Section 3.2.)

**distrib-20403** ☑ An **Uncertainty** object may contain one and only one instance of the **ListOfUncertParameters** element. No other elements from the SBML Level 3 Distributions namespaces are permitted on an **Uncertainty** object. (Reference: SBML Level 3 Specification for Distributions, Version 1, Section 3.10 on page 18.)

**distrib-20404** ☑ An **Uncertainty** object may have the optional attributes `distrib:id` and `distrib:name`. No other attributes from the SBML Level 3 Distributions namespaces are permitted on an **UncertSpan** object. (Reference: SBML Level 3 Specification for Distributions, Version 1, Section 3.10 on page 18.)

**distrib-20450** ☑ An **Uncertainty** object may have only one or zero **UncertParameter** children of each of the following types: "`distribution`", "`coeffientOfVariation`", "`kurtosis`", "`mean`", "`median`", "`mode`", "`sampleSize`", "`skewness`", "`standardDeviation`", "`standardError`", or "`variance`". (Reference: SBML Level 3 Specification for Distributions, Version 1, Section 3.10 on page 18.)

**distrib-20451** ☑ An **Uncertainty** object may have only one or zero **UncertSpan** children of each of the following types: "`confidenceInterval`", "`credibleInterval`", "`interquartileRange`" or "`range`". (Reference: SBML Level 3 Specification for Distributions, Version 1, Section 3.10 on page 18.)

#### *Rules for UncertSpan object*

**distrib-20501** ☑ An **UncertSpan** object may have the optional SBML Level 3 Core attributes `metaid` and `sboTerm`. No other attributes from the SBML Level 3 Core namespaces are permitted on an **UncertSpan**. (Reference: SBML Level 3 Version 1 Core, Section 3.2.)

**distrib-20502** ☑ An **UncertSpan** object may have the optional SBML Level 3 Core subobjects for notes and annotations. No other elements from the SBML Level 3 Core namespaces are permitted on an **UncertSpan**. (Reference: SBML Level 3 Version 1 Core, Section 3.2.)

**distrib-20503** ☑ An **UncertSpan** object must have the required attribute `distrib:type`, and may have the optional attributes `distrib:id`, `distrib:name`, `distrib:value`, `distrib:var`, `distrib:units` and `distrib:definitionURL`, `distrib:varLower`, `distrib:valueLower`, `distrib:varUpper` and `distrib:valueUpper`. No other attributes from the SBML Level 3 Distributions namespaces are permitted on an **UncertSpan** object. (Reference: SBML Level 3 Specification for Distributions, Version 1, Section 3.12 on page 21.)

**distrib-20504** ☑ The value of the attribute `distrib:varLower` of an **UncertSpan** object must be the identifier of an existing object derived from the ***SBase*** class and defined in the enclosing **Model** object. (Reference: SBML Level 3 Specification for Distributions, Version 1, Section 3.12 on page 21.)

**distrib-20505** ☑ The attribute `distrib:valueLower` on an **UncertSpan** must have a value of data type `double`. (Reference: SBML Level 3 Specification for Distributions, Version 1, Section 3.12 on page 21.)

**distrib-20506** ☑ The value of the attribute `distrib:varUpper` of an **UncertSpan** object must be the identifier of an existing object derived from the ***SBase*** class and defined in the enclosing **Model** object. (Reference: SBML Level 3 Specification for Distributions, Version 1, Section 3.12 on page 21.)

**distrib-20507** ☑ The attribute `distrib:valueUpper` on an **UncertSpan** must have a value of data type `double`. (Reference: SBML Level 3 Specification for Distributions, Version 1, Section 3.12 on page 21.)

**distrib-20550** ☑ An **UncertSpan** object may define either the attribute `distrib:valueLower` or `distrib:varLower`, but not both. (Reference: SBML Level 3 Specification for Distributions, Version 1, Section 3.11 on page 19.)

**distrib-20551** ☑ An **UncertSpan** object may define either the attribute `distrib:valueUpper` or `distrib:varUpper`, but not both. (Reference: SBML Level 3 Specification for Distributions, Version 1, Section 3.11 on page 19.)

**distrib-20552** ☑ The value of the attribute `distrib:type` of an **UncertSpan** object must be a subset of the allowed values of SBML data type `UncertType`, that is, the value must be one of the following: "`externalParameter`", "`confidenceInterval`", "`credibleInterval`", "`interquartileRange`" or "`range`". (Reference: SBML Level 3 Specification for Distributions, Version 1, Section 3.11 on page 19.)

**distrib-20553** ☑ An **UncertSpan** object with a `type` of "`confidenceInterval`", "`credibleInterval`", "`interquartileRange`" or "`range`" may not define the attributes `distrib:value`, `distrib:var`, or `distrib:definitionURL`. (Reference: SBML Level 3 Specification for Distributions, Version 1, Section 3.11 on page 19.)

**distrib-20554** ☑ An **UncertSpan** object with a `type` of "`confidenceInterval`", "`credibleInterval`", "`interquartileRange`" or "`range`" may not define a child **Math** or **ListOfUncertParameters** object. (Reference: SBML Level 3 Specification for Distributions, Version 1, Section 3.11 on page 19.)

## B Acknowledgments

Much of the initial concrete work leading to this proposal document was carried out at the Statistical Models Workshop in Hinxton in 2011, which was organized by Nicolas Le Novère. A list of participants and recordings of the discussion is available from http://sbml.org/Events/Other_Events/statistical_models_workshop_2011. Before that a lot of the ground work was carried out by Darren Wilkinson who led the discussion on *distrib* at the Seattle SBML Hackathon and before that Colin Gillespie who wrote an initial proposal back in 2005. The authors would also like to thank the participants of the *distrib* sessions during various HARMONY and COMBINE meetings for their excellent contributions in helping revising this proposal; Sarah Keating, Maciej Swat, Nicolas Le Novère, and Matthias König for useful discussions, corrections and review comments; and Mike Hucka for LaTeX advice, text editing, and the template upon which this document is based.
